# Development and characterization of protein kinase B/AKT isoform-specific nanobodies

**DOI:** 10.1371/journal.pone.0240554

**Published:** 2020-10-12

**Authors:** Tijs Merckaert, Olivier Zwaenepoel, Kris Gevaert, Jan Gettemans

**Affiliations:** 1 Department of Biomolecular Medicine, Faculty of Medicine and Health Sciences, Ghent University, Ghent, Belgium; 2 VIB-UGent Center for Medical Biotechnology, Ghent, Belgium; University of Hawai'i at Manoa, UNITED STATES

## Abstract

The serine/threonine protein kinase AKT is frequently over-activated in cancer and is associated with poor prognosis. As a central node in the PI3K/AKT/mTOR pathway, which regulates various processes considered to be hallmarks of cancer, this kinase has become a prime target for cancer therapy. However, AKT has proven to be a highly complex target as it comes in three isoforms (AKT1, AKT2 and AKT3) which are highly homologous, yet non-redundant. The isoform-specific functions of the AKT kinases can be dependent on context (i.e. different types of cancer) and even opposed to one another. To date, there is no isoform-specific inhibitor available and no alternative to genetic approaches to study the function of a single AKT isoform. We have developed and characterized nanobodies that specifically interact with the AKT1 or AKT2 isoforms. These new tools should enable future studies of AKT1 and AKT2 isoform-specific functions. Furthermore, for both isoforms we obtained a nanobody that interferes with the AKT-PIP3-interaction, an essential step in the activation of the kinase. The nanobodies characterized in this study are a new stepping stone towards unravelling AKT isoform-specific signalling.

## Introduction

The PI3K/AKT/mTOR pathway is one of the most frequently dysregulated pathways in cancer, up to 30% of human cancers have obtained mutations in one or several members of this pathway [1, 2]. One of its central nodes, the Ser/Thr protein kinase AKT (also known as Protein Kinase B), regulates multiple cellular processes, among them cell survival, metabolism, growth and proliferation [1]. Although AKT itself is rarely mutated, the proteins that regulate AKT such as PI3K, PTEN and PHLPP are frequently mutated, resulting in increased AKT activity, tumour cell growth and survival [3]. This made AKT an attractive target for the treatment of tumours with PI3K/AKT/mTOR pathway mutations [4, 5]. However, the development of small-molecule AKT inhibitors has long been hampered by the structural similarity of the AKT catalytic domain to that of other kinases of the AGC kinase group [5–7]. In addition, there are several known cases of ATP-competitive inhibitors that induce AKT hyperphosphorylation and activity [8]. To overcome such issues, the focus shifted towards the development of allosteric inhibitors (such as MK-2206, miransertib and AKT1/2), which show highly improved specificity towards AKT [5, 9, 10].

In recent years however, it has become clear that specificity for the AKT family is not sufficient [11]. The AKT kinase comes in three isoforms (AKT1, AKT2 and AKT3) each encoded by a separate gene [12]. All AKT isoforms share the same basic building blocks: an N-terminal Pleckstrin homology (PH) domain, a linker, catalytic domain and C-terminal regulatory domain [12]. In unstimulated cells the PH-domain keeps AKT in an inactive conformation (PH-in) through interaction with the catalytic domain. In response to upstream signalling–the interaction of the PH-domain with phosphatidylinositol (3,4,5)-trisphosphate (PIP3) produced by PI3K –AKT shifts to an open conformation (PH-out) enabling activation by phosphorylation on a threonine residue in the catalytic domain and a serine residue in the C-terminal regulatory domain [5, 13–15]. The isoforms are highly homologous with 82% sequence identity for AKT1 vs AKT2, 83% for AKT1 vs AKT3 and 77% for AKT2 vs AKT3. Despite their homology, they have non-redundant and, in certain cases, opposed functions [11, 16–24]. A striking example are the roles of AKT1 and AKT2 in breast cancer where AKT2 enhances migration and invasion, whereas AKT1 inhibits these processes [16, 19, 20, 25, 26]. This underlines the need for new tools to study AKT isoform-specific functions, which directly affect the protein, rather than the proteins’ expression levels. Such tools can aid in drug discovery or, through rational drug design, be the next step towards the development of an isoform-specific inhibitor [27]. Indeed, targeting a single AKT isoform to counteract the function of the drivers relevant to a specific type of cancer would alleviate the toxicity observed when using pan-AKT inhibitors and be beneficial for patient outcome [28].

The serum of alpacas and other members of the *camelidae* family contain Heavy-Chain-Only antibodies (HCAb). This subtype of IgG, discovered by the Hamers-Casterman group, completely lacks light chains (LC) and the first constant domain of the heavy chain (HC) [29]. The antigen-binding fragment of such a HCAb consists of a single domain that remains functional when isolated. This domain, called a variable domain of the heavy chain of a HCAb (VHH) or a nanobody (Nb), is approximately 15 kDa in size and measures 2.5 by 4 nm [30, 31]. Structurally, a Nb is composed of four well conserved framework regions (FR) and three hypervariable regions (HV), the latter forming loops that cluster at the N-terminal side of the folded Nb, where they form the antigen-binding surface or complementarity-determining region (CDR). The HV loops of a Nb are usually longer than those found in the variable domain of the heavy chain (VH), to compensate for the lack of the variable domain of the light chain (VL) which, in conventional Abs, forms the CDR together with the VH. The three HV loops of the Nb provide a CDR of 600–800 Å which, unlike the CDR of a conventional Ab, forms a convex surface [30–33]. In conventional Abs, the main chain atoms of the variable loops can only adopt a few different conformations, called canonical structures. However, the HV loops of a Nb are not limited to such canonical conformations [33] and therefore, Nbs can bind clefts on protein surfaces which cannot be targeted by conventional Abs [34]. As such, Nbs are small, stable, single-domain and high-affinity binders that can access cryptic epitopes[30]. Nbs can be used to target intracellular proteins and block specific protein functions [27, 32]. To achieve this, Nbs can be expressed intracellularly (intrabodies) using transfection or transduction. Alternatively, they can be introduced into the cell through photoporation, coupled to a cell-penetrating peptide or injected into cells by exploiting the *E*. *coli* T3SS [35–37].

To date there are no alternatives to genetic manipulation to study/interfere with the function of a single AKT isoform. The allosteric inhibitors, currently the most promising candidates for specific AKT inhibition, function by binding the PH domain and locking AKT in the PH-in conformation, preventing AKT activation[5, 10]. To obtain isoform-specific AKT nanobodies that interact with the AKT PH-domains, an alpaca was immunized with recombinant AKT1 PH-domain or the mutant form which confers constitutive membrane localization and activation of AKT1 (AKT1-E17K), full-length and activated AKT2 or the AKT3 PH-domain [13]. Using this approach, we obtained AKT1 and AKT2-specific binders, which can be expressed as intrabodies in mammalian cells. Furthermore, both for AKT1 and AKT2, a single specific Nb was obtained that interacts with the PH-domain and interferes with the AKT-PIP3-interaction *in vitro*. As AKT isoform-specific interactors, these Nbs offer new possibilities to study and interfere with the functions of these kinases at the protein level. As such, these new tools can be a step towards solving the complex roles of the AKT isoforms.

## Results

### Generation of AKT nanobodies

The AKT1-, AKT1-E17K- and AKT3-PH domains were expressed in BL21 *E*. *coli* cells, purified using TALON^®^-immobilized metal affinity chromatography (IMAC) (Clontech) and subsequent anion-exchange chromatography (MonoQ, GE Healthcare). Purity of the eluted fractions was assessed by SDS-PAGE. Purified and active AKT2 was purchased from Active Motif (S1 Fig).

Three separate VHH libraries were constructed: one for AKT1, the second for AKT1-E17K and AKT3, and the third for AKT2. The libraries for AKT1 and AKT2 were constructed using the pMECS phagemid vector and contained 5x10^7^ independent transformants (~87% with correct insert size) and 3x10^8^ independent transformants (~80% with correct insert size) respectively. For AKT1-E17K and AKT3, the pHEN4 phagemid vector was used and a library with 7.7x10^7^ independent transformants (~93% with correct insert size) was obtained. Panning (2 rounds for AKT1 and 4 rounds for AKT1-E17K, AKT2 and AKT3), enzyme-linked immunosorbent assay (ELISA) on crude periplasmatic extracts and sequencing of positive colonies yielded 17 AKT1 Nbs, 8 AKT1-E17K Nbs, 10 AKT2 Nbs and 11 AKT3 Nbs.

### Screening for AKT isoform-specific binders

**Fig 1:**
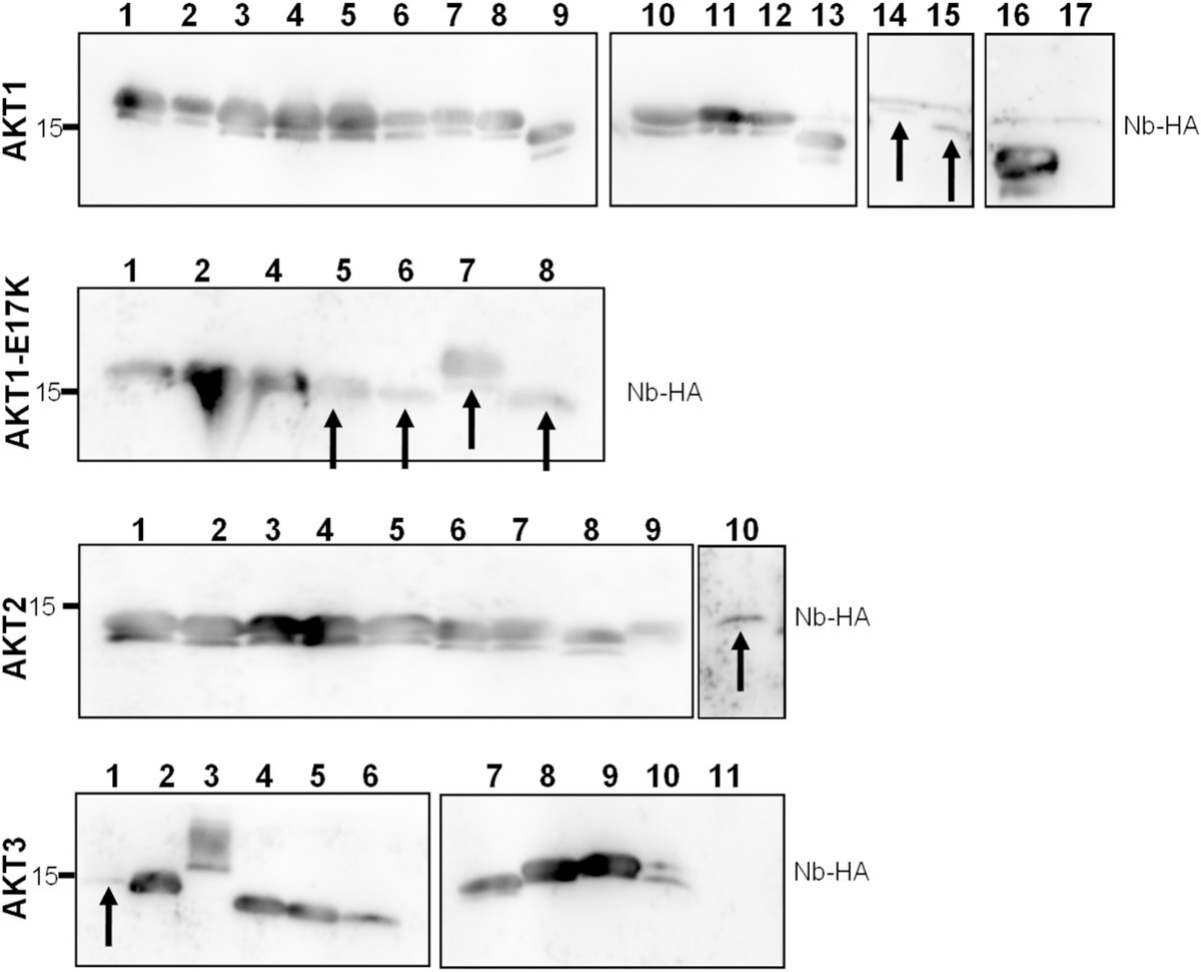
Expression of AKT nanobodies in WK6 *E*. *coli*. Western blot detection of AKT Nbs in a crude periplasmatic extract on a 15% SDS gel. An anti-HA Ab was used to detect the Nbs. The vast majority of the Nbs had comparable and high expression yields. The expression of AKT1 Nb14 and Nb15, AKT1-E17K Nbs5-8, AKT2 Nb10 and AKT3 Nb1 was low (denoted by an arrow) whereas only AKT1 Nb17 and AKT3 Nb11 could not be detected. Uncropped blots are available in S1 Raw images.

**Fig 2:**
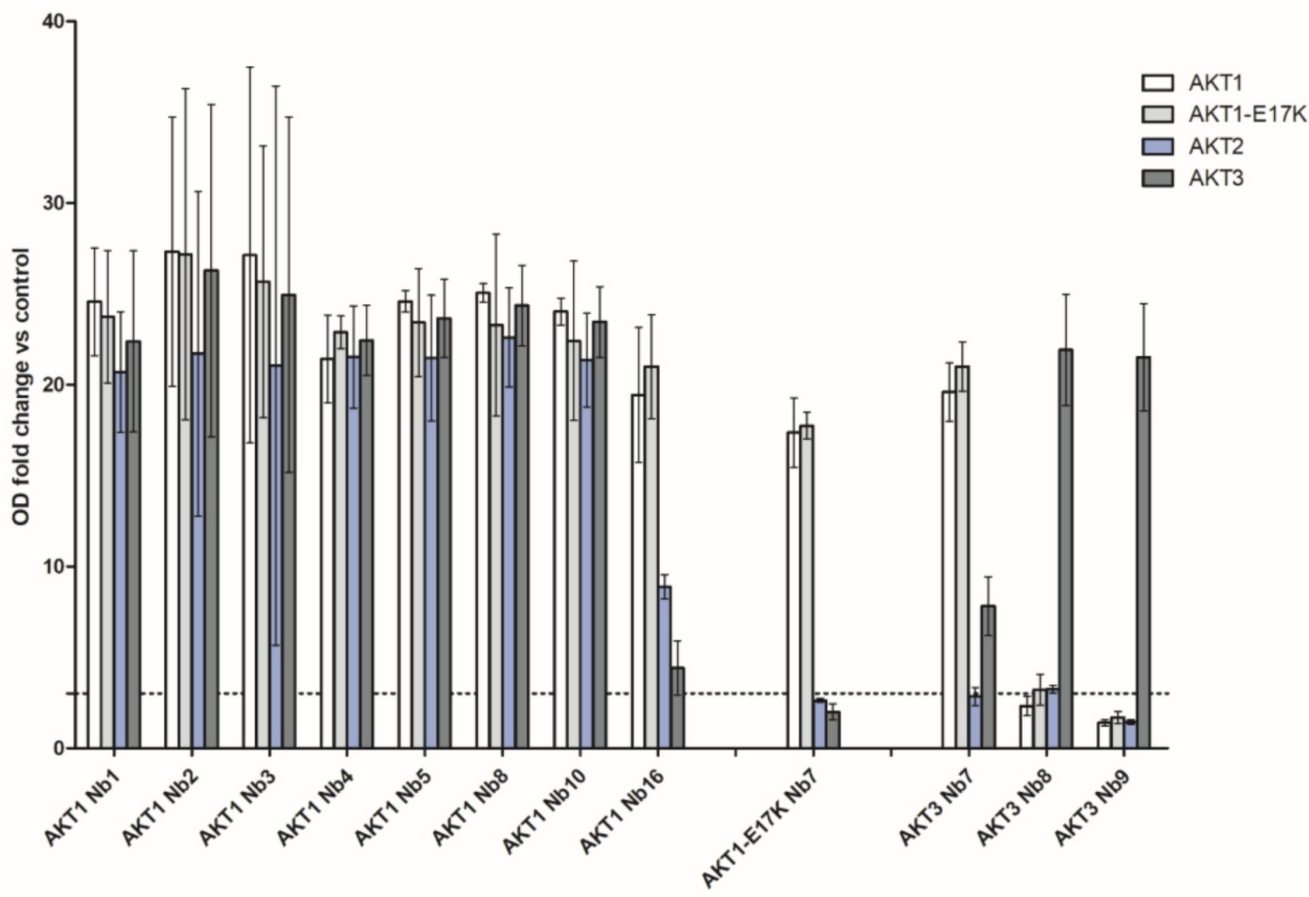
ELISA screening of AKT pleckstrin homology domain nanobodies. The mean and 95% confidence interval (CI) of OD_405_ values are shown. Pleckstrin Homology domains were coated in wells of a 96 multiwell plate at 1μg/ml and incubated with the AKT Nbs (20μl from a crude periplasmatic extract). The EGFP Nb was used for background correction. A Nb was considered to interact with a PH domain when the OD_405_ fold change (normalized to the OD_405_ of the EGFP Nb for the same PH domain) was at least three (denoted by a dashed line). Nbs, which did not meet this criterion for any PH domain are not shown on this Figure and were not included in further analysis. ELISA data for the complete Nb sets are available in S2 Fig.

Nb expression was assessed though Western blot analysis of WK6 *E*. *coli* crude periplasmatic extracts (Fig 1). Almost all Nbs were successfully expressed at high levels, Nbs with low expression levels were denoted by an arrow in Fig 1. Only two Nbs (AKT1 Nb17 and AKT3 Nb 11) yielded no signal, which indicated no expression. In the pMECS vector, the His_6_ tag is cleaved off upon storage of the Nb at 4°C this resulted in a second band with lower molecular weight that was also detected by the anti-HA Ab. The AKT1, AKT1-E17K and AKT3 Nb sets were screened for both strength of interaction and specificity through ELISA (Fig 2). In contrast to the ELISA performed during panning, where each library was screened for interaction with the PH-domain used for immunization, each Nb was tested for cross-reactivity with each of the AKT PH-domains (AKT1, AKT1-E17K, AKT2 and AKT3). A Nb was considered to interact with a PH-domain when the measured optical density (OD)_405_ was at least three times higher than that of the EGFP Nb (negative control) for the corresponding PH-domain (OD fold change > 3, denoted as dashed line in Fig 2) [38]. 8 AKT1 Nbs met this OD fold change requirement, however the results clearly indicated these Nbs did not specifically interact with AKT1. AKT1-E17K Nb7 interacted with both AKT1 and the oncogenic mutant AKT1-E17K, but showed no true preference for interacting with the mutant form of this PH-domain (p>0.05). AKT3 Nb7 interacted with AKT1, AKT1-E17K and AKT3, judging by the OD fold change, this Nb bound more strongly with both the wild type and mutant form of the AKT1 domain than with AKT3 (p<0.05). AKT3 Nb8 and Nb9 both interacted with AKT3 but not with the other AKT isoforms. It should be noted that, for AKT3 Nb8, the measured OD_405_ for the other PH-domains almost reached the OD fold change requirement for being considered an interactor. Results from this initial screening were used to select Nbs that have the greatest potential to be isoform-specific binders. The ELISA for the AKT1-, AKT1-E17K- and AKT3-Nbs indicated that we had obtained a single Nb specific for AKT1 (which binds both wild type and the E17K mutant) and two AKT3-specific Nbs. Additionally, AKT1 Nb8 was included in further screening experiments as pan-AKT Nb and AKT3 Nb7 as an AKT1 & AKT3 interactor.

**Fig 3:**
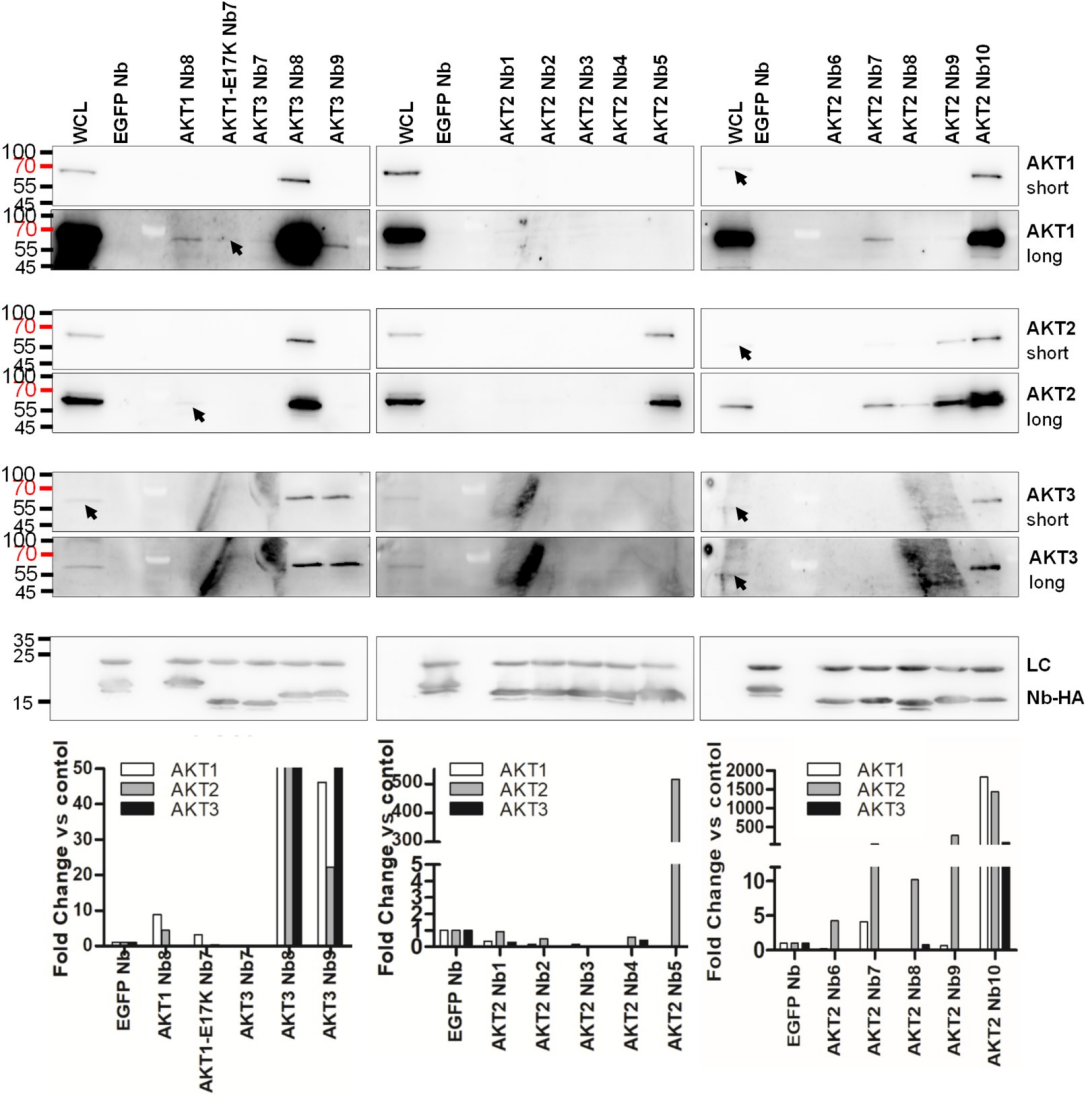
Co-IP of AKT isoforms using AKT1-, AKT1E17K-, AKT2- and AKT3-Nbs. Immunoprecipitation of endogenous AKT1, AKT2 or AKT3 from MDA-MB-231 cells with recombinantly produced HA-tagged Nbs. Representative for three repeated experiments. For the AKT isoforms a pair of panels is shown for each isoform with a short exposure time and a long exposure time. The short exposure panel contains no saturated pixels. WCL = whole cell lysate, EGFP Nb = negative control of anti-HA-agarose incubated with the EGFP Nb and MDA-MB-231 lysate and LC = Light-Chain. Nbs were blotted using an anti-HA antibody, AKT1, AKT2 and AKT3 were detected using the C73H10, D6G4 and 62A8 isoform-specific Ab clones respectively. All nanobodies (Nb-HA) were efficiently expressed in *E*. *coli*. Uncropped blots are available in S1 Raw images.

Interaction of these Nbs with endogenous full-length AKT1, AKT2 and AKT3 isoforms from MDA-MB-231 cells was determined through Co-Immunoprecipitation (Co-IP) using recombinantly produced hemagluttin (HA)-tagged Nbs. As the AKT2 Nbs were produced using full-length AKT2, the entire AKT2 Nb set was included in this analysis. Anti-HA coated beads were incubated with whole cell lysate (WCL) as negative control. As shown by the clear band at ~15 kDa, all Nbs were efficiently produced in WK6 *E*. *coli* and enriched on the anti-HA-agarose beads (Fig 3). Western blot analysis (Fig 3) of the Co-IP using AKT isoform-specific antibodies and quantification of the ECL signal showed AKT1 Nb8 to pull-down endogenous AKT1 and AKT2, but not AKT3. AKT1-E17K Nb7 pulled-down only AKT1. AKT2 Nbs 5, 6, 7, 8, 9 and 10 were able to pull-down AKT2. In addition to AKT2, AKT2 Nb7 cross-reacted with AKT1 and AKT2 Nb10 with both AKT1 and AKT3. Although AKT2 Nbs 1–4 were expressed to a similar extent, the signal for AKT was never higher than the control for any isoform. AKT3 Nb7 did not yield signal higher than the background for any AKT isoform. AKT3 Nb8 and Nb9 pulled-down AKT3 to a similar extent but both Nbs also interacted with AKT1 and AKT2.

Taken together with the ELISA screening, these results indicate immunization with the AKT1 PH domain did not yield Nbs specific for this isoform. We did, however obtain an AKT1-specific Nb that interacted with both the wild-type and E17K mutant. For AKT2, we obtained a set of 4 Nbs (AKT2 Nb5, Nb6, Nb8 and Nb9) that only interacted with AKT2 in a Co-IP and 2 Nbs (AKT2 Nb7 and Nb10) that interacted with several isoforms. Due to the low signal for AKT2, Nb6 was excluded from further experiments. No AKT3-specific Nbs were obtained as AKT3 Nb7 also interacted with AKT1 and AKT3 Nb8 and Nb9 bound all three isoforms in Co-IP experiments.

### Epitope mapping of AKT2 nanobodies

**Fig 4:**
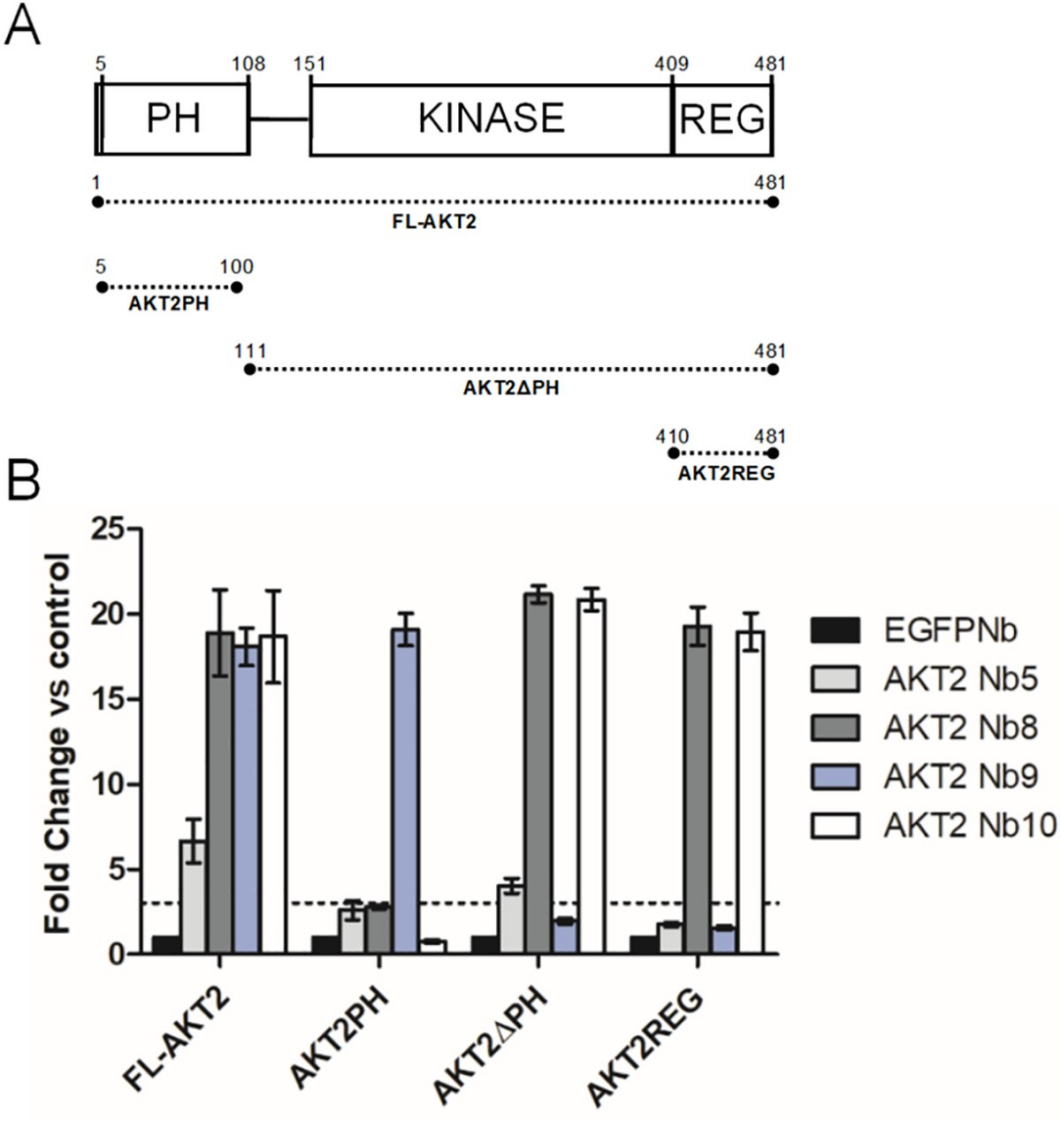
Epitope mapping of AKT2 Nbs. (A) Illustration of the AKT fragments used for epitope mapping. (B) ELISA: Mean and 95% CI of normalized OD plotted for full-length AKT2 (FL-AKT2), the AKT2 PH domain (AKT2PH), a fragment consisting of the flexible linker, kinase domain and regulatory domain (AKT2ΔPH) and the regulatory domain (AKT2REG). Target proteins were coated in wells of a 96 multiwell plate in quadruplicate for each AKT2 Nb and the EGFP Nb (negative control). All measured OD’s were normalized for the negative control. A Nb was considered to be an interactor when the average normalized OD was at least three times that of the negative control for the same AKT2 fragment (dashed line).

To narrow down the AKT2 domain(s) bound by the AKT2 Nbs, constructs were designed for recombinant expression of full-length AKT2 (FL-AKT2), the AKT2 PH-domain (AKT2PH), AKT2 lacking the PH-domain (AKT2ΔPH) and the C-terminal regulatory domain (AKT2REG) (Fig 4A). Nb-protein interaction was determined by ELISA, the EGFP Nb was used as a negative control (Fig 4B). AKT2 Nbs 5, 8, 9 and 10 interacted with FL-AKT2 with a fold change in normalized OD of 6.66±0.8, 18.9±1.6, 18.1±0.7 and 18.69±1.7 respectively. This indicated that, even though the Nbs were produced by immunization with active (phosphorylated) AKT2, the Nbs can also bind unphosphorylated AKT2, this being AKT2 in its inactive conformation. AKT2 Nb9 was the only Nb that interacted with the AKT2 PH-domain (OD fold change 19.1±0.6). AKT2 Nbs 5, 8 and 10 interacted with AKT2ΔPH (OD fold change of 4.02±0.27, 21.18±0.3 and 20.8±0.4 respectively). Both Nb8 and Nb10 also interacted with the regulatory domain (OD fold change of respectively 19.3±3.66 and 18.97±3.74) whereas Nb5 did not, suggesting Nb5s’ epitope to be located on the flexible linker, kinase domain or a region overlapping the boundary of the kinase- and regulatory domain.

It was previously shown that Nbs can be used as the primary antibody for detection of proteins through Western blotting [39]. This depends on the type of epitope the Nb interacts with (linear vs. conformational). None of the AKT Nbs were able to detect AKT in crude lysates from MDA-MB-231 cells, indicating that the Nbs interact with a conformational epitope which is lost on denaturation of AKT during sample preparation.

### AKT pleckstrin homology domain Nbs interfere with PIP3 interaction

**Fig 5:**
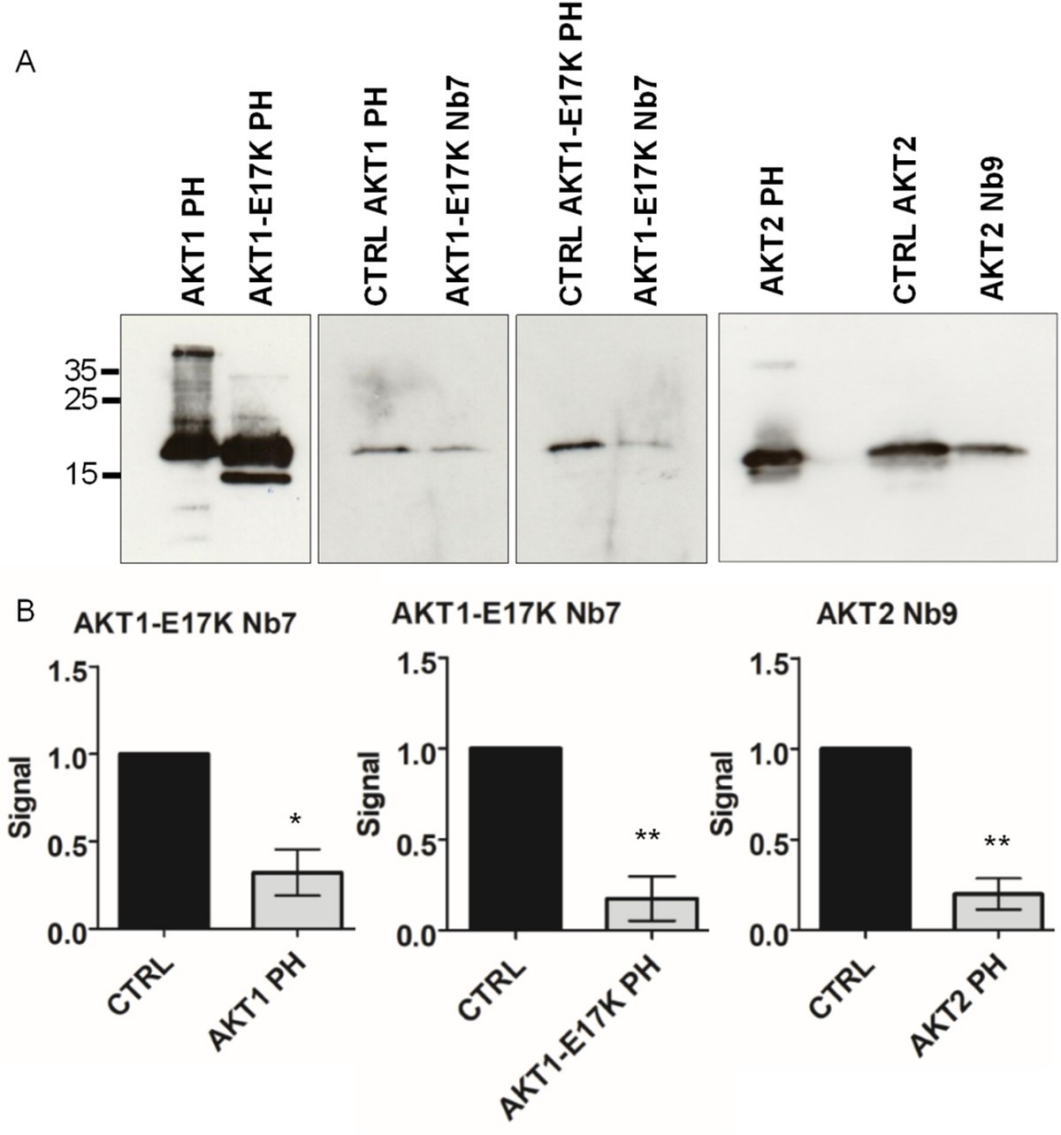
AKT Nanobodies interfere with AKT-PH—PIP3 interaction. (A) Western Blot of pull-down experiments using PIP_3_—coated beads and the AKT PH-domains. For each PH domain a positive control (CTRL) was included where the PH-domains were incubated with the beads without Nb. When the PH-domains were incubated with AKT1-E17K Nb7 or AKT2 Nb9 before PIP_3_-coated beads were added we observe a reduction in the signal for AKT1 PH- and AKT1-E17K PH- or AKT2 PH-domain respectively. Representative for three repeated experiments. *p≤0.05, **p≤0.01 (paired t-test). The AKT1-, AKT1-E17K-–and AKT2-PH-domain were detected using an Ab specific for AKT1 (C73H10) and AKT2 (D6G4) respectively. Uncropped blots are available in S1 Raw images. (B) ImageJ quantification of western blots. All signal intensities were normalized for the appropriate control (CTRL). The mean (bar) and SD (whiskers) are shown.

Interaction of the AKT Pleckstrin Homology domains with PIP3 is responsible for the membrane recruitment of the AKT isoforms, where they can be activated [14]. Isoform-specific Nbs that interact with the PH-domain (AKT1-E17K Nb7 and AKT2 Nb9) have the potential to interfere with this interaction, thereby inhibiting the activation of a single AKT isoform. A protein pull-down experiment using agarose beads coated with PIP3, recombinantly produced PH-domains and Nbs showed AKT1-E17K Nb7 interfered with the PIP3-PH-domain interaction for the PH-domains of AKT1 and AKT1-E17K and AKT2 Nb9 for AKT2 (Fig 5). When compared to the control conditions where only PH-domain and PIP3-beads were incubated (CTRL) we found that a pre-incubation of PH-domain and Nb significantly reduced (paired t-test) the amount of PH-domain pulled down by the PIP3-coated beads as determined by Western blot detection of the AKT PH-domains.

### Transient expression of AKT Nbs in mammalian cells

**Fig 6:**
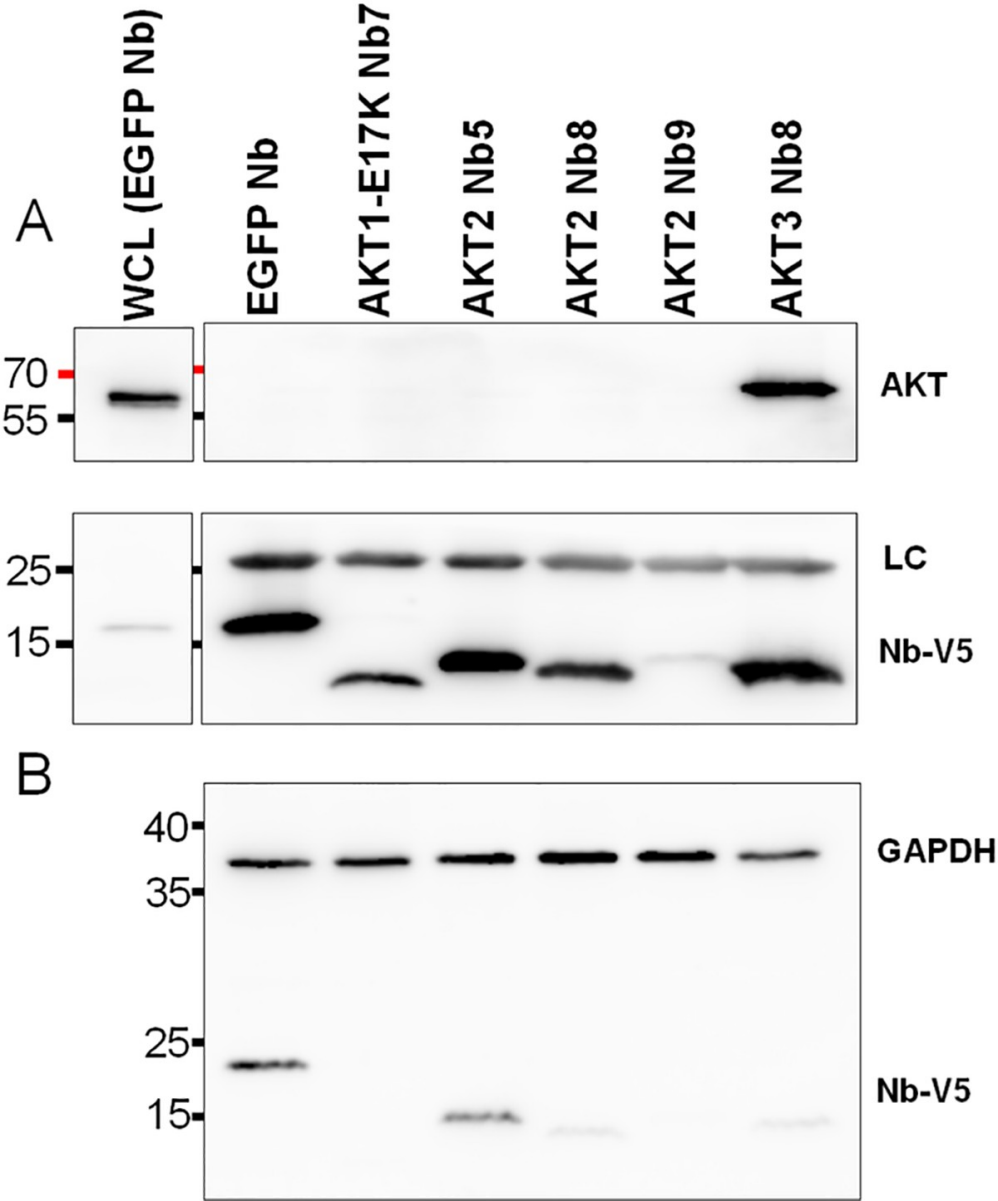
Co-IP of endogenous AKT from MDA-MB-231 cells with Nbs transiently expressed as intrabodies. (A) Co-IP of AKT and Nbs. WCL = whole cell lysate from EGFP Nb transfected cells, The negative control were cells with transient expression of the EGFP Nb. LC = Light Chain. (B) Nb expression in crude lysate. 10μg crude lysate from transfected cells was analysed through SDS-PAGE and western blotting. GAPDH signal was used as loading control. AKT was detected using a pan-AKT antibody (C67E7), Nbs were detected using an anti-V5-antibody. Uncropped blots are available in S1 Raw images.

MDA-MB-231 cells were transfected for transient expression of the AKT Nbs and the EGFP Nb, which served as negative control throughout this experiment. Analysis of the crude lysate (Fig 6B) indicated that not all Nbs were expressed to the same extent: the EGFP Nb displayed the highest expression levels closely followed by AKT2 Nb5. AKT2 Nb8 and AKT3 Nb8 clearly had lower expression levels (although the signal for the loading control for AKT3 Nb8 was lower as well), while AKT1-E17K Nb7 and AKT2 Nb9 could not be detected in crude lysate. Results from the Co-IP (Fig 6A) showed that AKT1-E17K Nb7 expression was detectable when enriched by the anti-V5-agarose, while the AKT2 Nb9 signal remained low under these conditions. The majority of the AKT Nbs was unable to pull-down any AKT, only AKT3 Nb8, which interacted with all three isoforms had a clear signal for AKT.

## Discussion

Targeting the AKT PH-domain has shown to be a promising strategy for interfering with AKT function: allosteric inhibitors such as MK-2206 and miransertib show superior selectivity for AKT over related kinases when compared to ATP-competitive inhibitors [5, 10, 40]. However, these inhibitors still target all AKT isoforms, which can be detrimental for cancer treatment considering the different and sometimes opposed functions of the AKT isoforms [4, 16]. In this study, we report the generation and characterisation of nanobodies specific for a single AKT isoform that interact with the PH-domain. Nb sets were generated by immunization of alpacas with the PH-domain of AKT1, AKT1-E17K (an oncogenic AKT1 mutant), the full-length and activated AKT2 and the AKT3 PH-domain. The obtained Nb sets were screened for specificity for a single AKT isoform through ELISA using recombinantly produced antigen and Co-IP using endogenous full-length AKT isoforms in crude lysate from MDA-MB-231 cells.

Due to the high degree of sequence identity for the AKT PH-domains, most of the obtained Nbs did not specifically interact with a single AKT isoform. The AKT1 Nbs interacted with all four PH-domains. A single representative (AKT1 Nb8) was included for the Co-IP where it pulled-down AKT1 and AKT2, but not AKT3. This deviation from the results observed in the ELISA could be explained by the much lower expression levels of AKT3 in MDA-MB-231 cells compared to AKT1 and AKT2 (unpublished results). The AKT3 Nb set did not yield any isoform specific Nbs. Even though the ELISA screening suggested AKT3 Nb9 to be AKT3-specific, Co-IP of the endogenous AKT isoforms did not corroborate this. It is possible that the particular epitope bound by these Nbs was no longer available when the AKT1-, AKT1-E17K- or AKT2-PH-domains were coated on the multiwell plate. As the main goal of this study was to identify isoform-specific Nbs that interact with the PH-domain, the AKT1 and AKT3 Nbs were omitted from further experiments.

AKT1-E17K Nb7 interacted with both the wild-type and mutant AKT1 PH-domains. Co-IP on endogenous AKT confirmed the AKT1-specificity. Although the signal for AKT1 was consistently higher than background it was relatively weak, with a fold change vs the EGFP Nb ranging from two to five. This indicated that binding of this Nb to the AKT1 PH-domain was not influenced by the conformational changes induced by the E17K mutation whereas this is known to confer resistance to the PH-domain targeting allosteric inhibitor Akti-1/2 and, although not linked to higher AKT1 activity, a decreased effect of MK-2206 on the phosphorylation of AKT1 S473 [41–43]. The AKT2 Nb set contained 6 Nbs that were able to pull-down AKT2 from MDA-MB-231 cells, four of those (Nbs 5, 6, 8 and 9) were specific for AKT2. This set yielded a single Nb that interacted with the AKT2 PH-domain (Nb9), one that interacted with the regulatory domain (Nb8) and one that bound the linker or kinase domain (Nb5).

Both AKT1-E17K Nb7 and AKT2 Nb9 could, for their respective AKT isoforms, interfere with the interaction between the recombinant PH-domain and PIP3-coated beads in an *in vitro* assay. For AKT1-E17K Nb7 this includes both the wild-type PH-domain and the oncogenic mutant. These Nbs, when expressed as intrabodies in cells, can potentially interfere with the activation of AKT1 or AKT2: AKT bound by one of these Nbs would be stuck in an inactive ‘PH-in’ conformation [15].

However, when transiently expressed in mammalian cells, we observed relatively low expression levels for all of the AKT Nbs. In a Co-IP experiment of AKT and the transiently expressed Nbs, we were only able to detect AKT for AKT3 Nb8, which interacted with all three isoforms and had already shown to be an exceptional binder in a Co-IP using recombinant Nb. It is highly likely that the low expression levels of the Nbs lie at the base of the lack of signal for the other tested Nbs. Although this is in our experience exceptional, it is also possible that the correct folding of the Nbs is affected by the reducing cytoplasmic environment, resulting in a loss-of-function. The low intracellular expression levels are a potential limitation to using these Nbs to study or interfere with specific AKT isoforms. These Nbs could benefit from alternative strategies to introduce the proteins into mammalian cells such as cell penetrating peptides, photoporation or electroporation [37].

Apart from interfering with AKT functions directly, an AKT isoform-specific Nb can be transformed into a tool to study protein function. Through the use of delocalization tags, a Nb can displace a target from its natural environment or when linked to a cullin-RING E3 ubiquitin ligase, the Nbs interaction with its target can trigger degradation of the target as an elegant alternative to RNAi [32, 44]. A co-crystal structure of the Nb and its target can also be used for structure-based drug design and lead to the first true AKT isoform-specific inhibitor [45–47].

In this study, we generated and characterized AKT1 and AKT2 isoform-specific binders (Table 1) that can be made into research tools to study the isoform-specific functions of the AKT kinase. Additionally, two of these Nbs have shown to interfere with the AKT activation mechanism in an *in vitro* assay.

**Table 1:** Summary of characteristics for relevant AKT nanobodies.

*Nanobody*	*Specificity (Co-IP)*	*Epitope (AKT domain)*	*Relative expression levels (MDA-MB-231)*	*Interferes with PH-PIP3 interaction*
***1PHE17K Nb7***	AKT1	PH	Medium	Yes
***2FL Nb5***	AKT2	Linker/ catalytic	High	N/A
***2FL Nb8***		Regulatory	Medium	N/A
***2FL Nb9***		PH	Low	Yes
***3PH Nb8***	AKT1,2,3	PH	Low	N/A

The Co-IP of AKT using recombinant Nbs was used as final criterium for specificity. For the AKT2 set the Nbs’ epitope was determined through ELISA, other Nb sets were generated by immunization with the PH-domain. Relative transient expression levels of the AKT Nbs in MDA-MB-231 cells was evaluated though Western blotting. AKT1-E17K Nb7 and AKT2 Nb2 interfere with the interaction the PH domain with PIP3 for the AKT1-, AKT1E17K- and AKT2PH-domain respectively. A summary of the characterization for the full Nb sets can be found in S1 and S2 Tables.

The high prevalence of AKT over-activation in cancer makes this kinase a high-profile target for cancer therapy. The Nbs obtained in this study are isoform-specific binders for AKT1 or AKT2. These can be made into research tools to investigate the isoform-specific functions of the AKT kinases in cells, offering alternatives to genetic approaches that will enable us to target a single AKT isoform. Additionally, two of these Nbs have shown to interfere with the AKT activation mechanism in an *in vitro* assay. These tools offer new opportunities to study the isoform-specific functions of AKT1 or AKT2 –and could lead to the first isoform-specific inhibitor.

## Materials and methods

### Immunization, library construction and panning

#### AKT-PH domain production

Plasmids containing constructs coding for HA-AKT1, Myc-AKT1-E17K, HA-AKT2 and HA-AKT3 were kind gifts from Donghwa Kim (H. Lee Moffit Cancer Center and Research Institute). cDNA encoding the PH-domains were isolated by PRC amplification using the following primers: Akt1PH Forward (Fwd) 5’ AGC GAA TTC ATG AGC GAC GTG GCT ATT GTG 3’, Akt1PH Reverse (Rev) 5’ GAA GCT TTC AGT TGT CAC TGG GTG AGC CCG ACC G 3’, Akt2PH Fwd AGC GAA TTC ATG AAT GAG GTG TCT GTC ATC 3’, Akt2PH Rev 5’ GAA GCT TTC ACA TCC ACT CCT CCC TCT CGT CTG G 3’, Akt3PH Fwd 5’ AGC GAA TTC ATG AGC GAT GTT ACC ATT GTG 3’ and Akt3PH Rev 5’ GAA GCT TTC AAT TCA TTC TCT CCT CTT CTT GCC TCT GC 3’. Constructs were inserted into a pHEN6 vector backbone using the Cold Fusion™ cloning kit (System Biosciences) according to the manufacturers’ protocol and proteins were expressed in BL21 *E*. *coli*.

#### Immunization and panning

Nbs were generated in collaboration with the VIB Nanobody Core as described in ‘Generation of single domain antibody fragments derived from camelids and generation of manifold constructs’ from Antibody Engineering: Methods and Protocols, second edition (2012). In short, alpacas were injected subcutaneously with the antigens on days 0, 7, 14, 21, and 35. Each injection contained 160 μg of protein for AKT1PH, 135 μg for AKT1PHE17K, 115 μg for AKT2FL and 250 μg for AKT3PH. Anticoagulated blood was collected on day 39. mRNA was extracted from lymphocytes, a VHH library was constructed and subjected to phage-display to select antigen-binding Nbs. All animal work was performed at the VIB Nanobody Core. Immunizations and animal handling was performed according to directive 2010/63/EU of the European parliament for the protection of animals used for scientific purposes and approved by the Ethical Committee for Animal Experiments of the Vrije Universiteit Brussel (permit No. 13-601-1). The alpacas used in this study were not euthanized as part of the study.

### In vitro nanobody characterization

#### Expression of recombinant AKT nanobodies

Heat Shock-competent WK6 *E*. *coli* (strain created by Dr. Gholamreza Hassanzadeh-Ghassabeh Nanobody Service Facility, VIB) were transformed with the AKT Nb constructs in a pMECS-(AKT2 and AKT1) or pHEN4-vector backbone (AKT1-E17K and AKT3) and grown overnight (ON) at 37°C on a lysogeny broth (LB 10 g/L tryptone, 5 g/L yeast extract, 5 g/L NaCl)-agar plate containing 100 **μ**g/mL Ampicillin. A single colony was picked and grown ON at 37°C in a shaking incubator in LB with 100 **μ**g/ml Ampicillin. The next day, 1/100 (v/v) was transferred to Terrific Broth (TB 16.9 mM KH_2_PO_4_, 71.9 mM K_2_HPO_4_.3H_2_O, 2 mM MgCl_2_, 12 g Tryptone, 24 g yeast extract, 4% glycerol, 1% glucose pH 7.2), grown to OD_600_ = 0.6–0.9, induced with 1 mM isopropylβ-D-thiogalactoside (IPTG) and incubated ON at 28°C in a shaking incubator. The next day, cultures were centrifuged at 4°C, 3,000 x g for 20 min. Nbs constructs in the pMECS- or pHEN4-vector are equipped with an N-terminal PelB signal sequence resulting in export of the Nbs to the periplasmatic space of *E*. *coli* and can be extracted through osmotic shock. Periplasmatic extracts were prepared by re-suspending the pellet in Tris-EDTA-sucrose buffer (TES) (0.2 M Tris-HCl, 0.5 mM EDTA and 0.5 M sucrose pH 8) and incubating for 1 h on ice with gentle agitation after which TES buffer diluted 4x in Milli-Q grade water was added and incubated for an additional hour (with shaking). Extracts were centrifuged at 4°C, 29,000 x g for 20 min and the supernatant (SN) was collected.

#### ELISA

A 96-multiwell plate (Nunc Maxisorp, Sigma-Aldrich) was coated overnight (ON) at 4°C with AKT1-, AKT1-E17K-, AKT2- or AKT3-PH-domains at 1 **μ**g/mL in Coating buffer (100 mM NaHCO_3_, pH 8.4). In between each of the subsequent steps, the plate was washed three times with 0.1% Tween in Phosphate Buffered Saline (PBS) (-Ca^2+^/Mg^2+^). The remaining protein-binding sites were blocked by incubation with 0.1% (w/v) Casein in PBS for 1 h at room temperature (RT). Periplasmatic extract from WK6 *E*. *coli* containing a Nb was diluted in PBS (-Ca^2+^/Mg^2+^) and 100 **μ**l was added to a well in quadruplicate for each for each PH-domain. After 1 h incubation at RT, each well was incubated (1 h at RT) with 100 **μ**l 0.5 **μ**g/mL anti-HA (#901502, BioLegend) antibody (Ab) and subsequently (1 h at RT) with 100 **μ**l 0.5 **μ**g/mL alkaline phosphatase (AP)-linked anti-mouse Ab (A90-116AP, Bethyl Laboratories). The reaction was initiated by adding 2 mg/mL AP substrate (4-nitrophenyl phosphate disodium salt hexahydrate, Sigma) in AP Blot Buffer (100 mM Tris-HCl, 50 mM MgCl_2_.6H_2_O, 100 mM NaCl, pH 9.50). Absorbance was measured at 405 nm. When the measured absorbance for an AKT Nb was at least threefold higher than for the EFGP Nb, the AKT Nb was considered to interact with that particular PH-domain. Significant differences in normalized OD_405_ were detected using a One-way ANOVA with Tukey’s test using GraphPad Prism V5.00.

#### Co-immunoprecipitation of endogenous AKT isoforms with recombinant nanobodies

Nbs were produced and extracted as described above. Anti-HA-agarose beads (A2095, Sigma Aldrich) were incubated with 10 μg periplasmatic extract for 1 h at 4°C with end-over-end rotation. The beads were washed with ice-cold Tris Lysis Buffer (20 mM Tris-HCl, 150 mM NaCl, 1% Triton X-100 pH 7.5), 1mM phenyl-metylsulfonyl fluoride (PMSF) and 200 μg/mL protease inhibitor cocktail) and incubated for 1 h at 4°C with end-over-end rotation with 1 mg crude lysate from MDA-MB-231 cells. A negative control where the EGFP Nb was added to the beads was included to determine non-specific binding of AKT to the agarose matrix and nanobody. The beads were washed three times with excess Tris Lysis buffer and heated to 95°C for 5 min in Laemmli Sample Buffer (5% SDS, 20% glycerol, 0,2% bromophenol blue, 5% β-mercaptoethanol, 65 mM Tris-HCl pH 6.8). Samples were analyzed by SDS-PAGE and Western blotting. The AKT isoforms were detected using isoform-specific Abs (AKT1 C73H10, AKT2 D6G4 and AKT3 62A8 from Cell Signaling Technology®) and the Nbs with an anti-HA-tag Ab (11583816001, Merck). ECL signal for the AKT isoforms was recorded using the Amersham Imager AI680 and the signal was quantified using Image Studio Lite (v5.2).

#### Epitope mapping of AKT2 nanobodies

Nanobodies were produced as described in “Expression of recombinant AKT nanobodies”. The AKT2 PH-domain was produced as described above, the other AKT2 fragments were created through PCR amplification based on the HA-AKT2 template using the following primers: FL-AKT2 Fwd 5’ TGT ACA GAA TGC TGG TCA TAT GAA TGA GGT GTC TGT CAT C 3’and FL-AKT2 Rev 5’ TCA CCC GGG CTC GAG GAA TTC TCA CTC GCG GAT GCT GGC CGA GTA GG 3' for full-length AKT2, AKT2ΔPH Fwd 5’ TGT ACA GAA TGC TGG TCA TAT GAA GCA GCG GGC CCC AGG CG 3’ and FL-AKT2 Rev for AKT2ΔPH and AKT2Reg Fwd 5’ GTA CAG AAT GCT GGT CAT ATG CTC AGC ATC AAC TGG CAG 3’ and FL-AKT2 Rev for the AKT2 Regulatory domain. PCR product was cloned into the pTYB12 vector using the Cold Fusion™ cloning kit (System Biosciences) according to the manufacturers’ protocol. BL21 *E*. *coli* were heat-shock transformed with the AKT2 constructs in a pTYB12 vector backbone. A culture was grown in TB to an OD_600_>2, expression of the constructs induced with 0.5 mM IPTG and the culture was incubated ON at 20°C in a shaking incubator. The following day, the cultures were centrifuged (20 min at 3000 x g, 4°C) and re-suspended in Chitin column buffer (20 mM Tris-HCl, 500 mM NaCl and 1 mM EDTA, pH 8.5) with 1 mM PMSF and 200 **μ**g/mL protease inhibitor cocktail. Cells were lysed using a French Press and sonication. Debris was pelleted by centrifugation (20 min at 29,000 x g, 4°C), supernatant was collected and loaded onto a column with Chitin beads (New England Biolabs) and allowed to empty by gravity flow at approximately 1 ml/min. Beads were washed with column buffer and incubated ON with column buffer containing 50 mM dithiothreitol (DTT). Elutions were collected and analysed by SDS-PAGE and Coomassie staining. The ELISA epitope mapping was performed as described above with the AKT2 constructs coated in the wells and AKT2 Nbs (in crude periplasmatic extract) added in solution.

### PIP3 pull-down

Nbs from a periplasmatic extract were purified using TALON™-IMAC (Clontech) according to the manufacturers’ protocol. In short, periplasmatic extracts were incubated for 2 h at 4°C with end-over-end rotation with TALON™ resin pre-equilibrated with wash buffer (50 mM NaH_2_PO_4_, 500 mM NaCl and 20mM imidazole, pH 8). The resin was washed three times with wash buffer and bound proteins were eluted with elution buffer (50 mM NaH_2_PO_4_, 500 mM NaCl and 500 mM imidazole, pH 8). Purity of the proteins was evaluated using SDS-PAGE and Coomassie staining.

For the pull-down experiment, a Nb was incubated at a 10X molar excess with PH-domain in Binding Buffer (10 mM HEPES, 150 mM NaCl and 0.25% NP-40, pH 7.4) for 1 h at 4°C with end-over end rotation. Subsequently 50 μl PIP3-coated beads (P-B345A, Echelon Biosciences) were added to the mixture. A negative control containing only beads and a PH-domain was also included at this point. After a 3 h incubation at 4°C with end-over-end rotation, the beads were washed three times with Binding Buffer and bound proteins were eluted by adding Laemmli sample buffer and heating the beads to 95°C for 5 min. Proteins were separated by size through SDS-PAGE followed by Western Blot analysis. The recombinant PH-domains of AKT1 and AKT2 were detected using an isoform-specific Ab (AKT1 C73H10 and AKT2 D6G4, Cell Signaling Technology®). Signal intensity was quantified using ImageJ, values were normalized for the positive control (no Nb). A paired t-test (GraphPad Prism v5.00) was used to determine significant changes in signal intensity.

### *In vivo* AKT nanobody properties

#### Subcloning Nbs for expression in mammalian cells

Nbs were subcloned to the pcDNA3.1 V5/His_6_ vector for transient expression in mammalian cells. PCR amplification of the Nbs was performed using pcDNA3.1 Fwd 5ʹTTG GTA CCG AGC TCG GCC ACC ATGCAG GTG CAG CTG CAG GAG 3ʹ and pcDNA3.1 Rev 5ʹTAG ACT CGA GCG GCC GCT GGA GAC GGT GAC CTG 3ʹ. Cloning was performed with the Cold Fusion™ cloning kit (System Biosciences) according to the manufacturers’ protocol.

#### Cell culture, transfection & pull-down

MDA-MB-231 (ATCC® HTB-26™) cells were cultured in Dulbecco’s Modified Eagle Medium (DMEM, Gibco, Thermo Fisher Scientific) supplemented with 10% fetal bovine serum (Gibco, Thermo Fisher Scientific), 100 IU/mL penicillin and 10 μg/mL streptomycin (Gibco, Thermo Fisher Scientific) [48]. Cells were grown in a humidified incubator at 37°C and 10% CO_2_. This cell line was obtained from Prof. Dr. De Wever (University of Ghent) in October 2011. Prior to experiments, the cell line was tested and found negative for mycoplasma contamination using PlasmoTest™ (Invivogen). For transient expression of the AKT Nbs, the JetPrime® transfection reagent (Polyplus Transfection) was used according to the manufacturers’ recommendations. 24 h after transfection, the cells were collected using Trypsin Ethylenediaminetetraacetic acid (EDTA, 0.05%, Gibco, Thermo Fisher Scientific) and lysed in ice-cold Tris Lysis Buffer. 1 mg crude extract was incubated with 10 μl settled anti-V5-agarose beads (A7345, Sigma Aldrich) for 1 h at 4°C with end-over-end rotation. The beads were washed three times with Tris Lysis Buffer and bound proteins were eluted by adding Laemmli sample buffer and heating the samples to 95°C for 5 min. Proteins were separated using SDS-PAGE and analyzed by Western Blotting. AKT was detected using a pan-AKT Ab (C67E7, Cell Signaling Technology) and the Nbs through their V5-tag with an anti-V5 Ab (R960-25, Thermo Fisher Scientific).

## Supporting information

S1 Checklist (PDF)

S1 Raw images (PDF)

S1 Fig Antigens for immunization. Uncropped gels. From left to right 1μg and 2μg of the AKT1 Pleckstrin homology domain (AKT1), the oncogenic mutant ATK1 PH-domain (AKT1-E17K), 1μg, 2μg and 5μg of full-length AKT2 (AKT2) and the AKT3 PH-domain (AKT3) respectively.
(TIF)

S2 Fig ELISA screening for AKT isoform-specific binders. Mean and 95% CI of OD_405_ values are plotted for the complete AKT1PH (A), AKT1PHE17K (B), and AKT3PH (C) nanobody sets. A dashed line shows the OD fold change requirement for a Nb to be considered an interactor for that particular PH domain.
(TIF)

S3 Fig Schematic representation of the Nb constructs. PelB = Signal sequence (22 amino acids) which directs the protein to the *E*. *coli* periplasm. This enables the release of recombinantly produced Nbs through osmotic shock. His_6_ = polyhistidine tag of 6 sequential histidine residues. This tag enables efficient purification of Nbs through IMAC but can also be used for detection. HA = human influenza hemagglutinin tag, a 9 amino acid (YPYDVPDYA) tag. V5 = derived from an epitope found in a virus from the SV5 family, 14 amino acid residues (GKPIPNPLLGLDST).
(TIF)

S1 Table Overview of the characterization for AKT1 and AKT1-E17K Nb sets. Summary of characterization for the AKT1- and AKT1-E17K- nanobody sets. Expression of the Nbs in WK6 *E*. *coli* was evaluated though SDS-PAGE & Western blot analysis of crude periplasmatic extracts. AKT isoform-specificity was assessed though both ELISA using recombinant AKT PH-domains (1 = AKT1 PH-domain, 1M = AKT1-E17K PH-domain, 2 = AKT2 PH-domain and 3 = AKT3 PH-domain) and a Co-IP using recombinant Nbs and the endogenous AKT isoforms from MDA-MB-231 crude lysates. A grey filled cell in the ‘ELISA specificity’ column indicates this Nb did not meet the OD fold change requirement for any PH-domain, these Nbs are not included in further experiments. The Co-IP was used as final criteria for specificity. Nbs that interact with an AKT PH-domain can interfere with the PIP3 interaction required for AKT activation. Using PIP3 coated beads, recombinant AKT PH-domains and Nbs we determined AKT1-E17K Nb7 interferes with the interaction of the AKT1 PH-domain AND AKT1-E17K PH-domain with PIP3. Using transient expression a selection of Nbs was expressed in mammalian cells (MDA-MB-231) and evaluated as intrabodies through a Co-IP of endogenous AKT.
(TIF)

S2 Table Overview of the characterization for AKT2 and AKT3 Nb sets. Summary of characterization for the AKT2- and AKT3-nanobody sets. Expression of the Nbs in WK6 *E*. *coli* was evaluated though SDS-PAGE & Western blot analysis of crude periplasmatic extracts. AKT isoform-specificity was assessed though both ELISA using recombinant AKT PH-domains (1 = AKT1 PH-domain, 1M = AKT1-E17K PH-domain, 2 = AKT2 PH-domain and 3 = AKT3 PH-domain) and a Co-IP using recombinant Nbs and the endogenous AKT isoforms from MDA-MB-231 crude lysates. A grey filled cell in the ‘ELISA specificity’ column indicates this Nb did not meet the OD fold change requirement for any PH-domain, these Nbs are not included in further experiments. The AKT2 Nbs were not included in the ELISA screening. The Co-IP was used as final criteria for specificity. AKT2 Nbs were produced by immunization with full-length AKT2, an ELISA epitope mapping was performed to determine which domain(s) these Nbs bind (PH = pleckstrin homology domain, LINK/KINASE = Linker and kinase domain, REG = regulatory domain). Nbs that interact with an AKT PH-domain can interfere with the PIP3 interaction required for AKT activation. AKT2 Nb9 interferes with the interaction of the AKT2 PH-domain with PIP3. Using transient expression a selection of Nbs was expressed in mammalian cells (MDA-MB-231) and evaluated as intrabodies through a Co-IP of endogenous AKT.
(TIF)
