# Supplementary material for: Development and characterization of protein kinase B/AKT isoform-specific nanobodies
Source: PLoS One. 2020 Oct 12;15(10):e0240554. doi: 10.1371/journal.pone.0240554 (PMC7549812; doi:10.1371/journal.pone.0240554)
Supplement: S1 Raw images — (PDF) [file pone.0240554.s002.pdf]

# S1_raw_images for

## Development and characterization of Protein Kinase B/AKT isoform-specific Nanobodies

Tijs Merckaert[1,2], Olivier Zwaenepoel[1], Kris Gevaert[1,2], Jan Gettemans*[1]

[1]Department of Biomolecular Medicine, Faculty of Medicine and Health Sciences, Ghent University, Ghent, Belgium

[2]VIB-UGent Center for Medical Biotechnology, Ghent, Belgium

*Corresponding author

E-mail: jan.gettemans@ugent.be

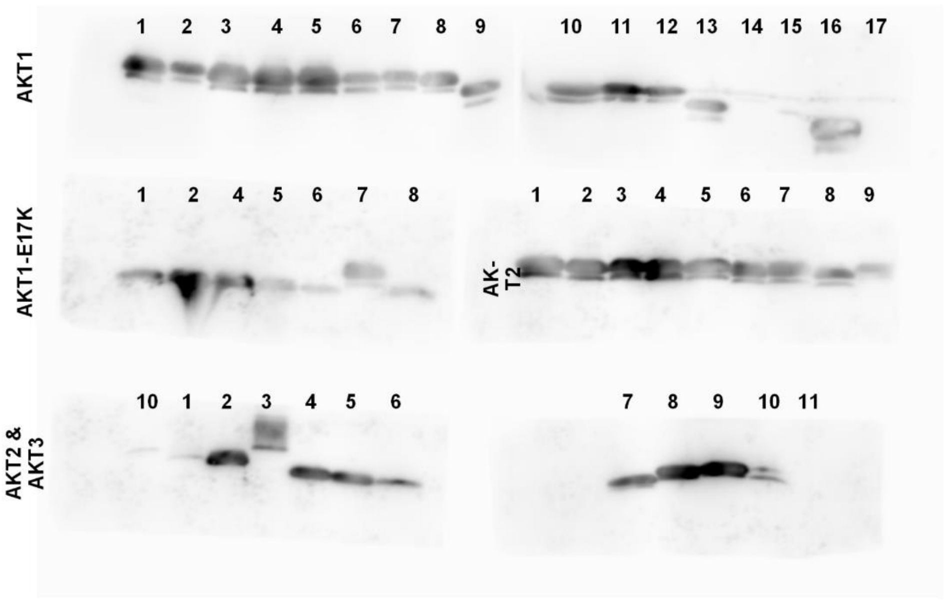
1 2 3 4 5 6 7 8 9 10 11 12 13 14 15 16 17
AKT1
1 2 4 5 6 7 8 1 2 3 4 5 6 7 8 9
AKT1-E17K
AK-T2
10 1 2 3 4 5 6 7 8 9 10 11
AKT2 & AKT3

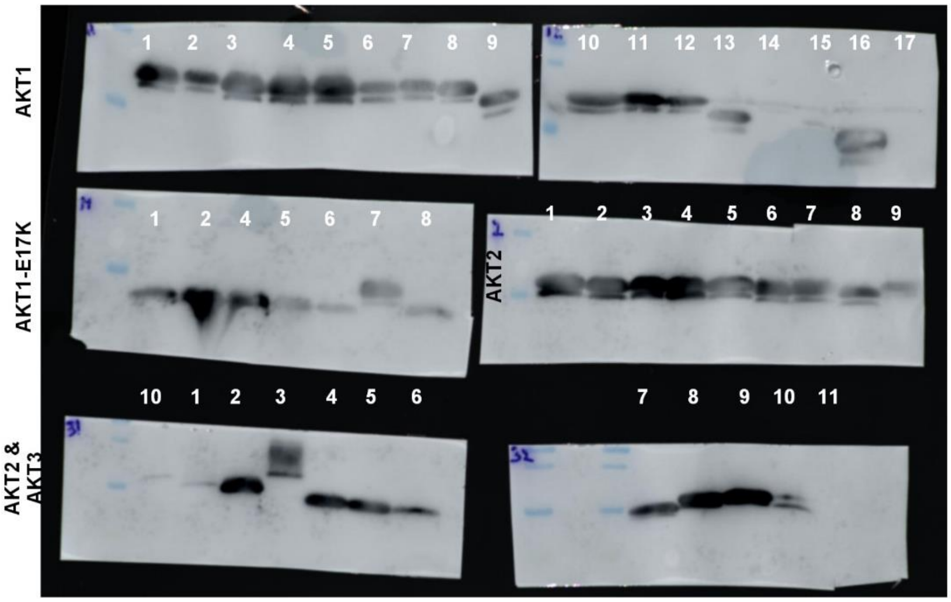
1 2 3 4 5 6 7 8 9 10 11 12 13 14 15 16 17
AKT1
1 2 4 5 6 7 8 1 2 3 4 5 6 7 8 9
AKT1-E17K
AKT2
10 1 2 3 4 5 6 7 8 9 10 11
AKT2 & AKT3

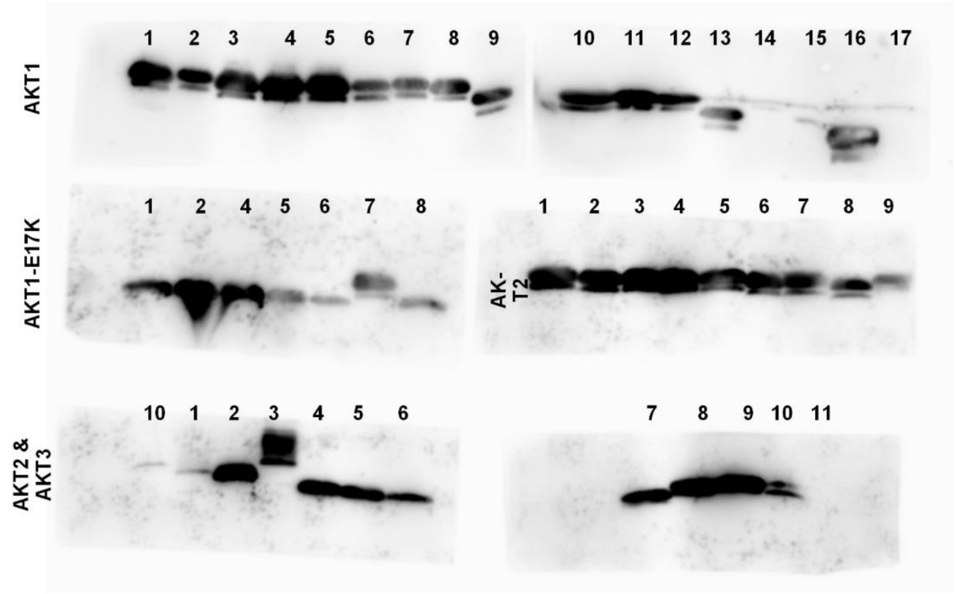


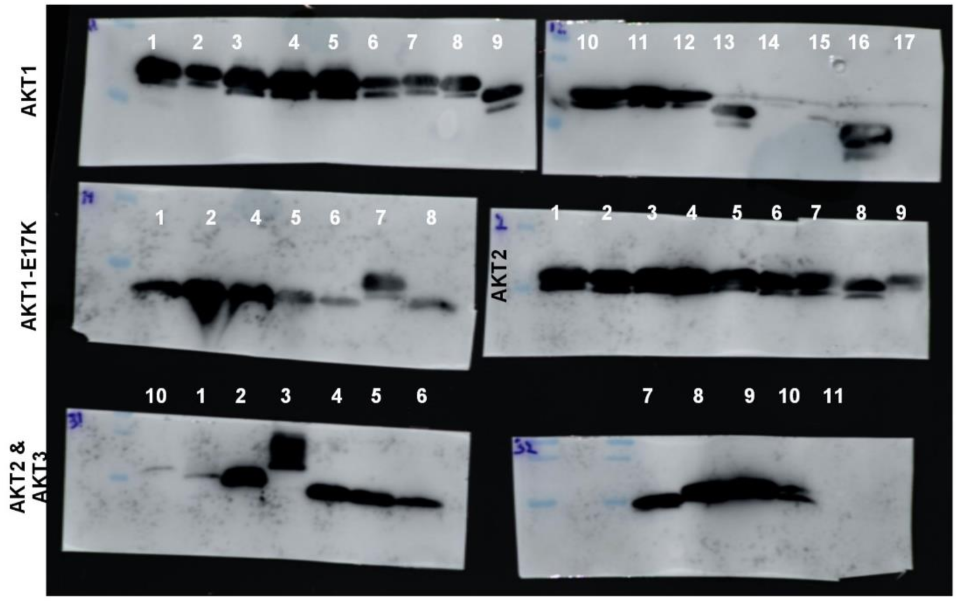


**Raw images for Fig 1: Expression of AKT nanobodies in WK6 *E. coli*.** Uncropped Western blot images for Fig1 recorded using the Amersham Imager 680 blot and gel imager. AKT nanobodies present in the crude periplasmatic extract were detected through enhanced-chemiluminescence using an anti-HA Ab and a secondary HRP-linked anti-mouse Ab. Nb signal is present at approximately 15kDa. Membranes are labelled with the library antigen and lanes with the Nb clone number.

# AKT1

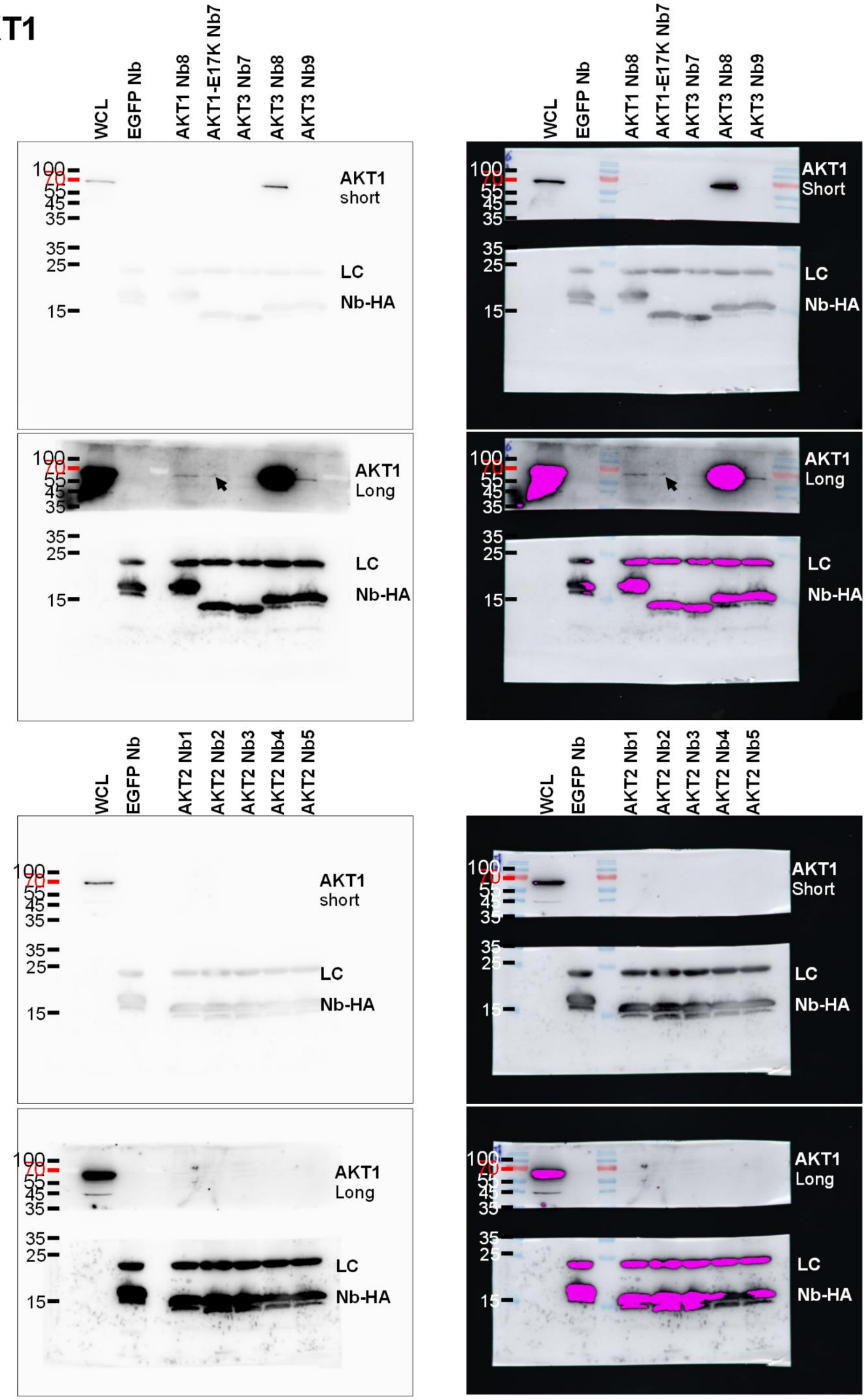

WCL
EGFP Nb
AKT1 Nb8
AKT1-E17K Nb7
AKT3 Nb7
AKT3 Nb8
AKT3 Nb9
100
70
55
45
35
25
15
AKT1 short
AKT1 Short
LC
Nb-HA
AKT1 Long
AKT2 Nb1
AKT2 Nb2
AKT2 Nb3
AKT2 Nb4
AKT2 Nb5

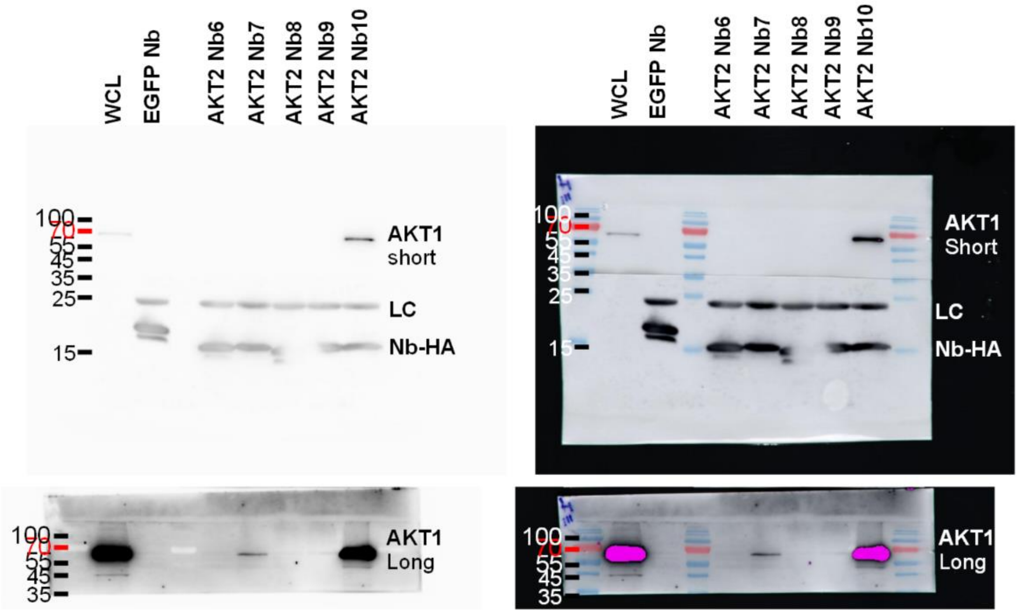
WCL
EGFP Nb
AKT2 Nb6
AKT2 Nb7
AKT2 Nb8
AKT2 Nb9
AKT2 Nb10
100
70
55
45
35
25
15
AKT1
short
LC
Nb-HA
AKT1
Short
AKT1
Long

## AKT2

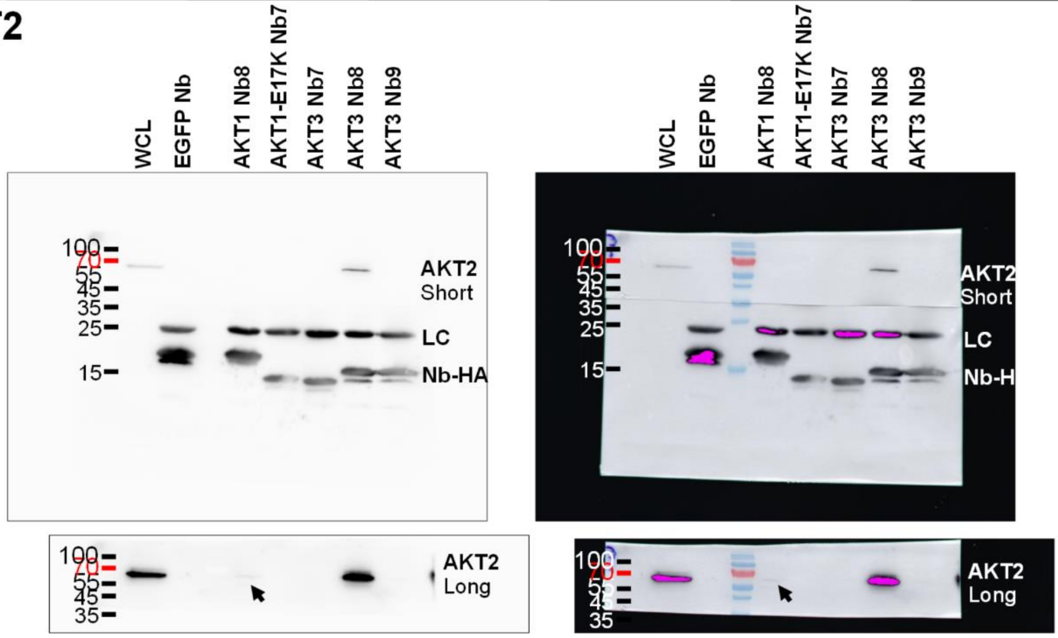
WCL
EGFP Nb
AKT1 Nb8
AKT1-E17K Nb7
AKT3 Nb7
AKT3 Nb8
AKT3 Nb9
100
70
55
45
35
25
15
AKT2
Short
LC
Nb-HA
Nb-H
AKT2
Long

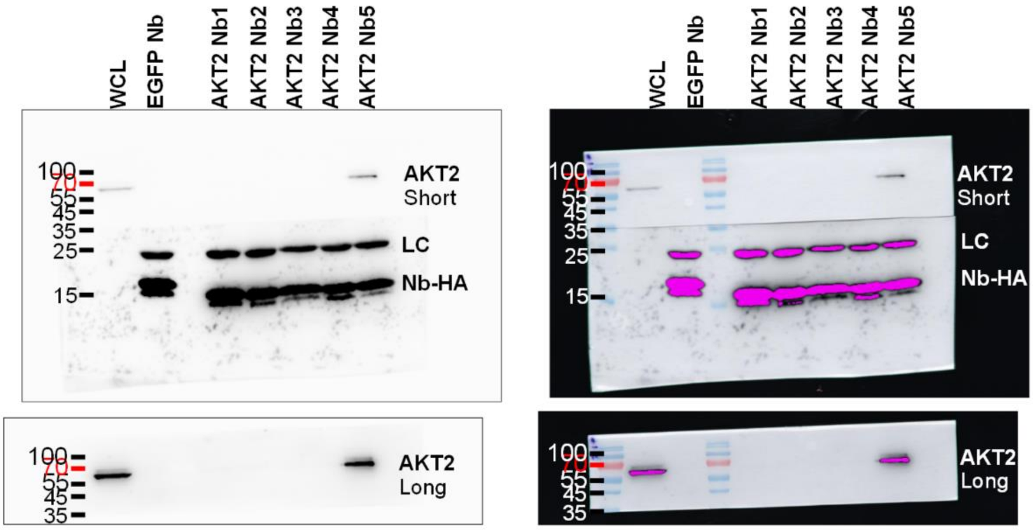
WCL
EGFP Nb
AKT2 Nb1
AKT2 Nb2
AKT2 Nb3
AKT2 Nb4
AKT2 Nb5
100
70
55
45
35
25
15
AKT2
Short
LC
Nb-HA
AKT2
Long

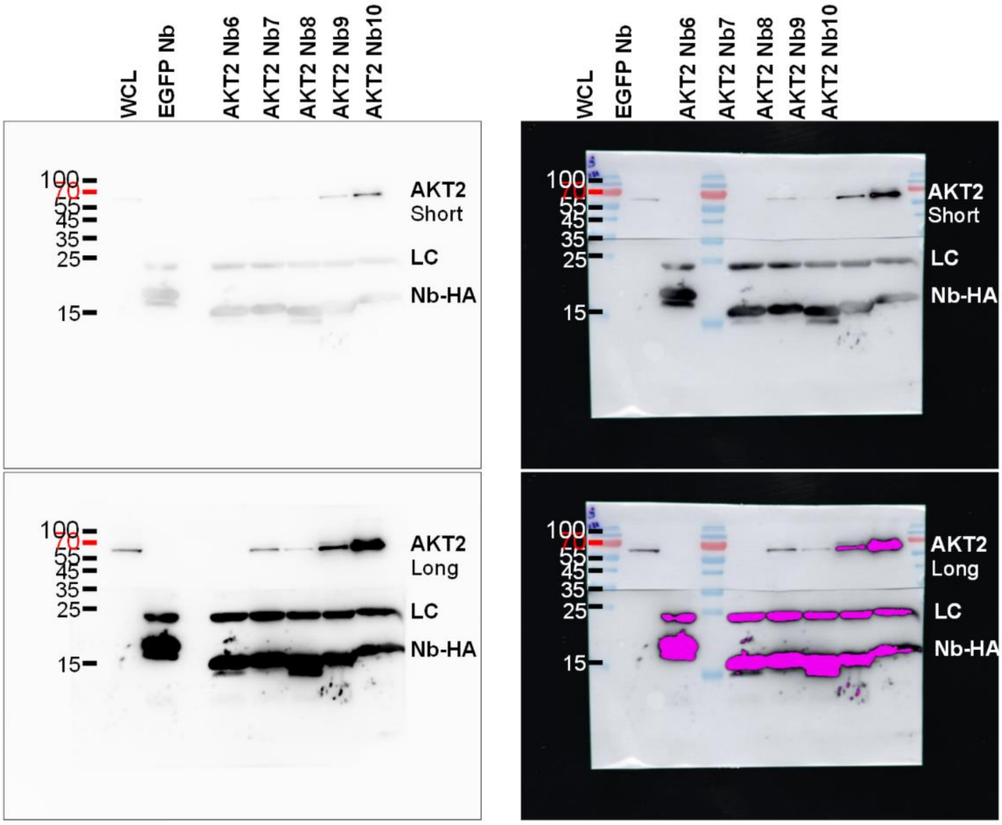
WCL
EGFP Nb
AKT2 Nb6
AKT2 Nb7
AKT2 Nb8
AKT2 Nb9
AKT2 Nb10
100
70
55
45
35
25
15
AKT2
Short
LC
Nb-HA
AKT2
Long
LC
Nb-HA

**AKT3**

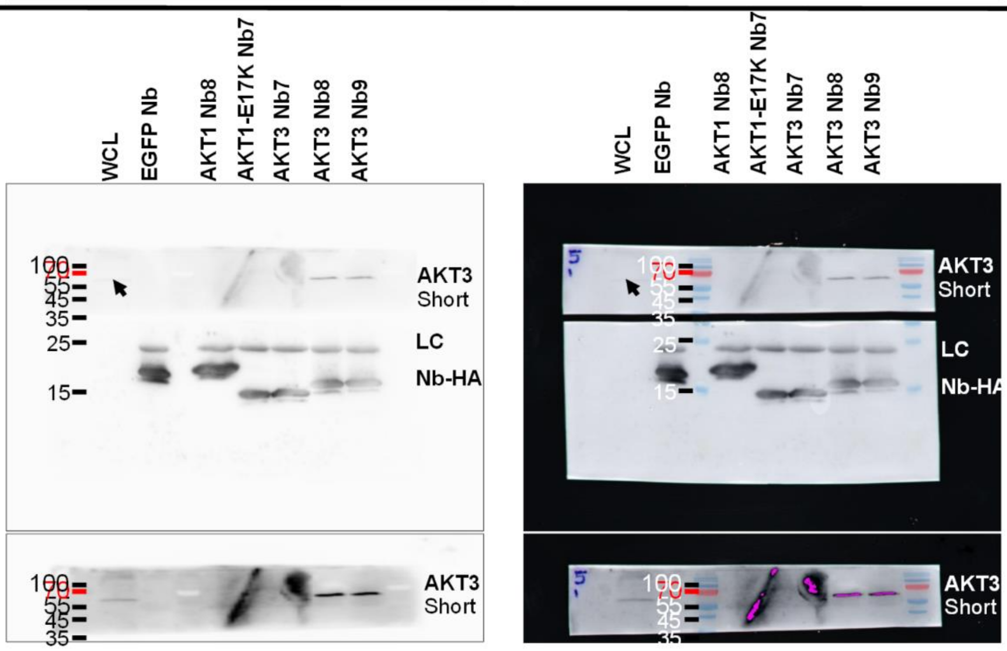
WCL
EGFP Nb
AKT1 Nb8
AKT1-E17K Nb7
AKT3 Nb7
AKT3 Nb8
AKT3 Nb9
100
70
55
45
35
25
15
AKT3
Short
LC
Nb-HA
AKT3
Short

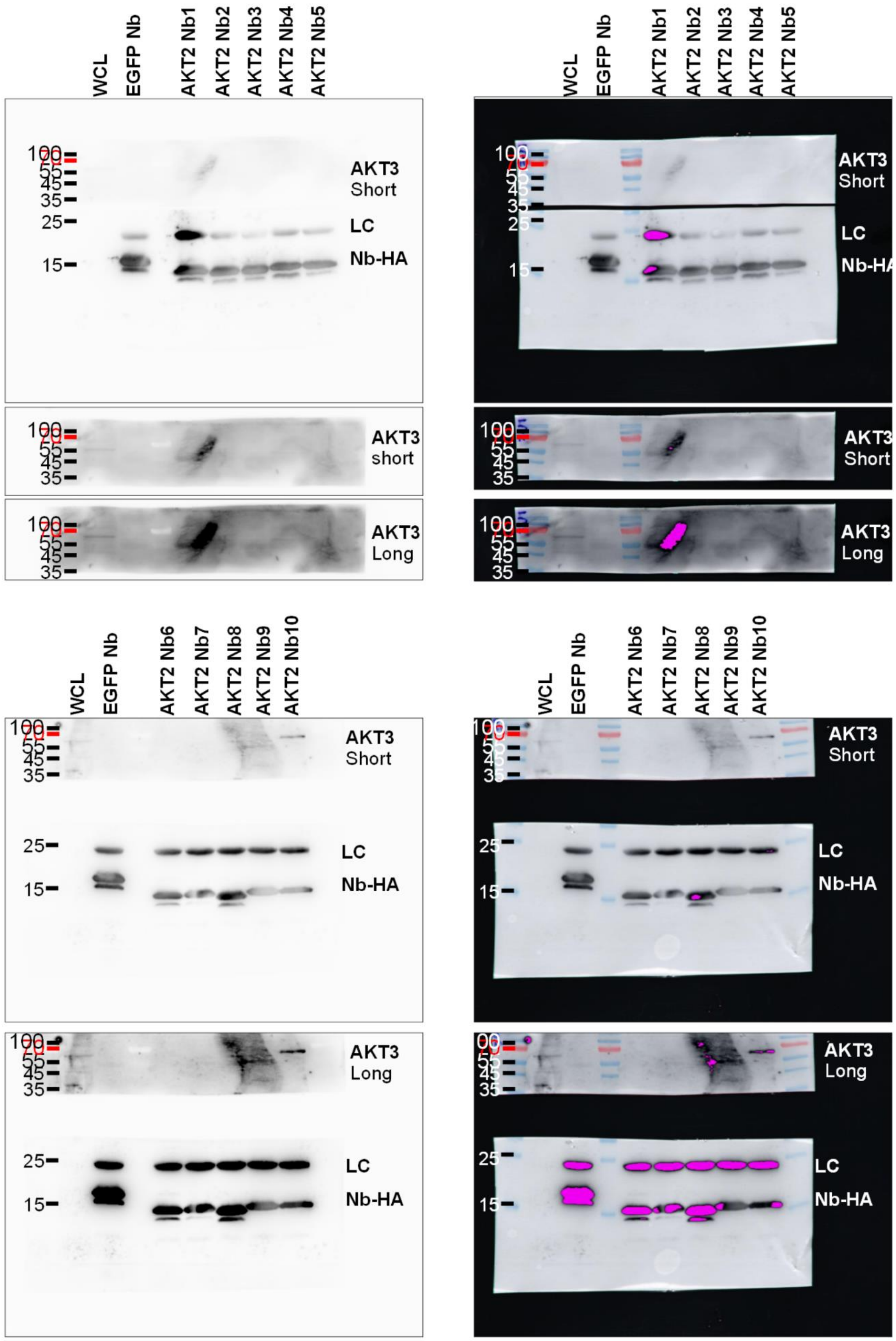


**Raw images for Fig 3: Co-IP of AKT isoforms using AKT1-, AKT1-E17K, AKT2 and AKT3-Nbs.**

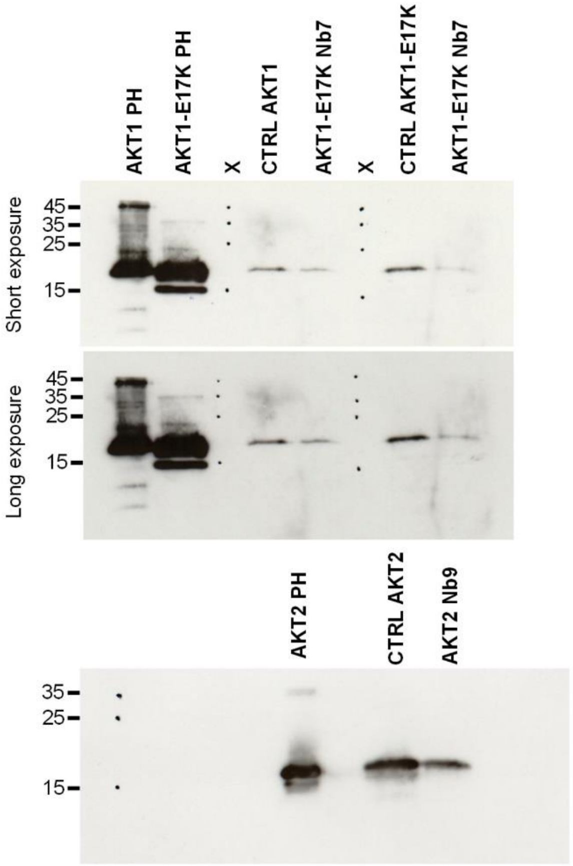


**Raw images for Fig 5: AKT nanobodies interfere with AKT-PH-PIP3 interaction.** Uncropped western blots for Fig5, pull-down experiments using PIP3-coated beads.

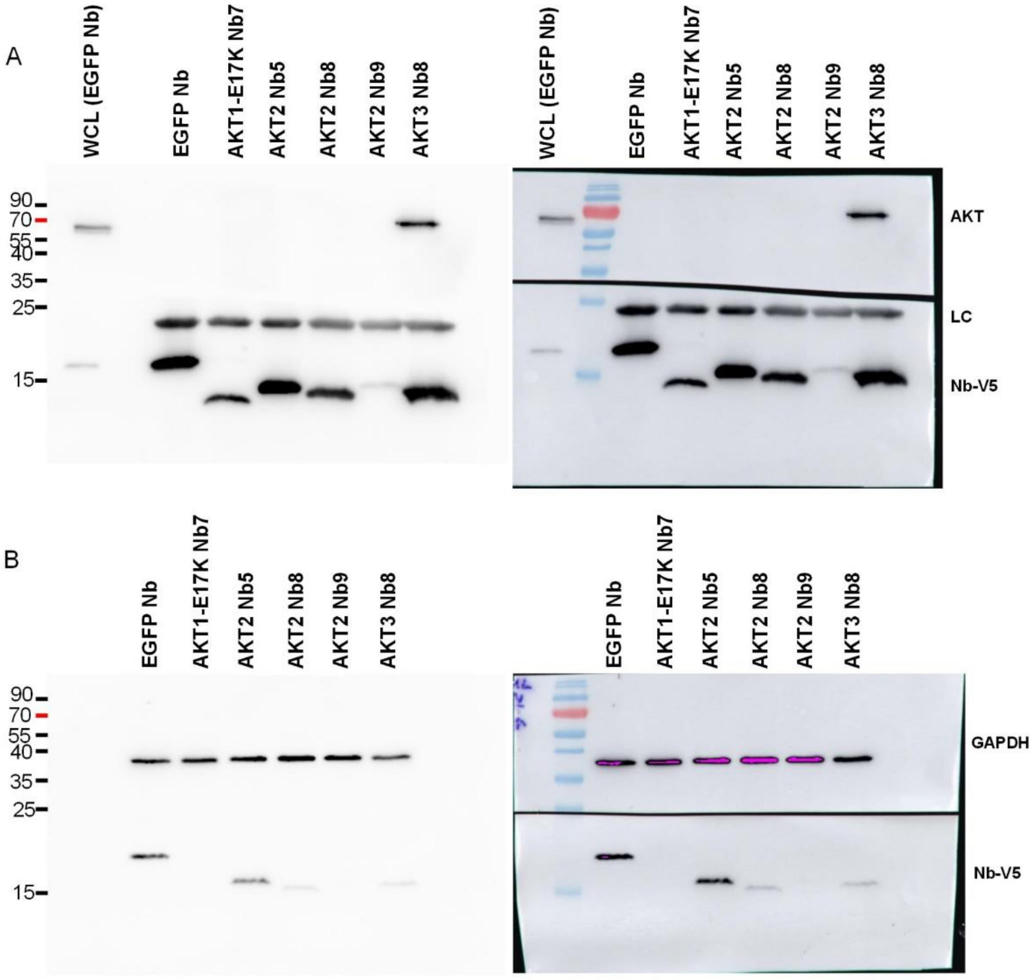


**Raw images for Fig6: Co-IP of endogenous AKT from MDA-MB-231 cells with Nbs transiently expressed as intrabodies.** Raw images for Fig 6. (A) Co-IP of AKT and Nbs. (B) Nb expression. Images were recorded using the Amersham Imager 680 blot and gel imager, the ECL signal is shown on the left. The right half of the figure shows the ECL signal superimposed on a photo of the blot membranes.
