# Supplementary figures and images for: Development and characterization of protein kinase B/AKT isoform-specific nanobodies
Source: PLoS One. 2020 Oct 12;15(10):e0240554. doi: 10.1371/journal.pone.0240554 (PMC7549812; doi:10.1371/journal.pone.0240554)

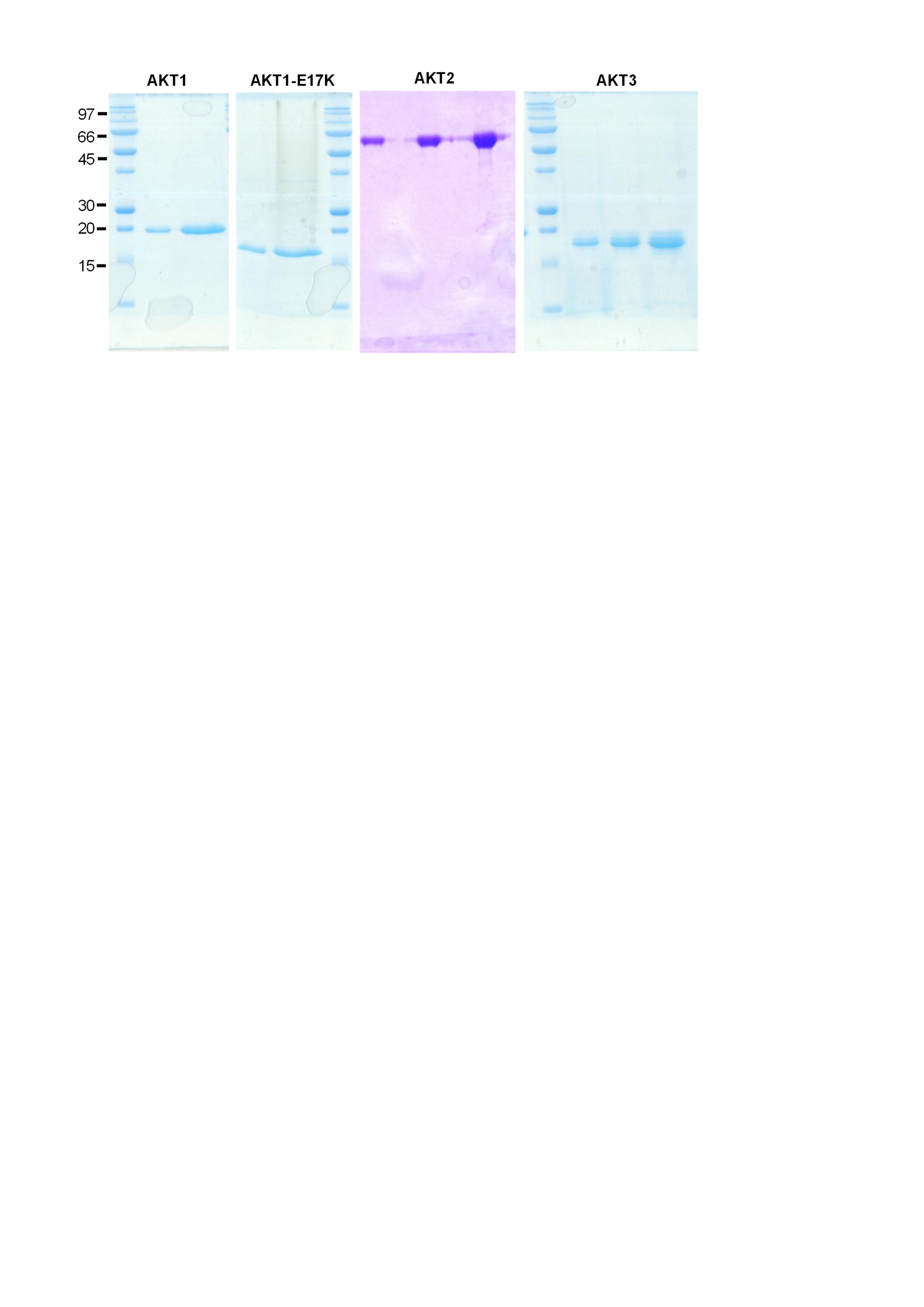

Supplement: S1 Fig — Uncropped gels. From left to right 1μg and 2μg of the AKT1 Pleckstrin homology domain (AKT1), the oncogenic mutant ATK1 PH-domain (AKT1-E17K), 1μg, 2μg and 5μg of full-length AKT2 (AKT2) and the AKT3 PH-domain (AKT3) respectively. (TIF) [file pone.0240554.s003.tif]

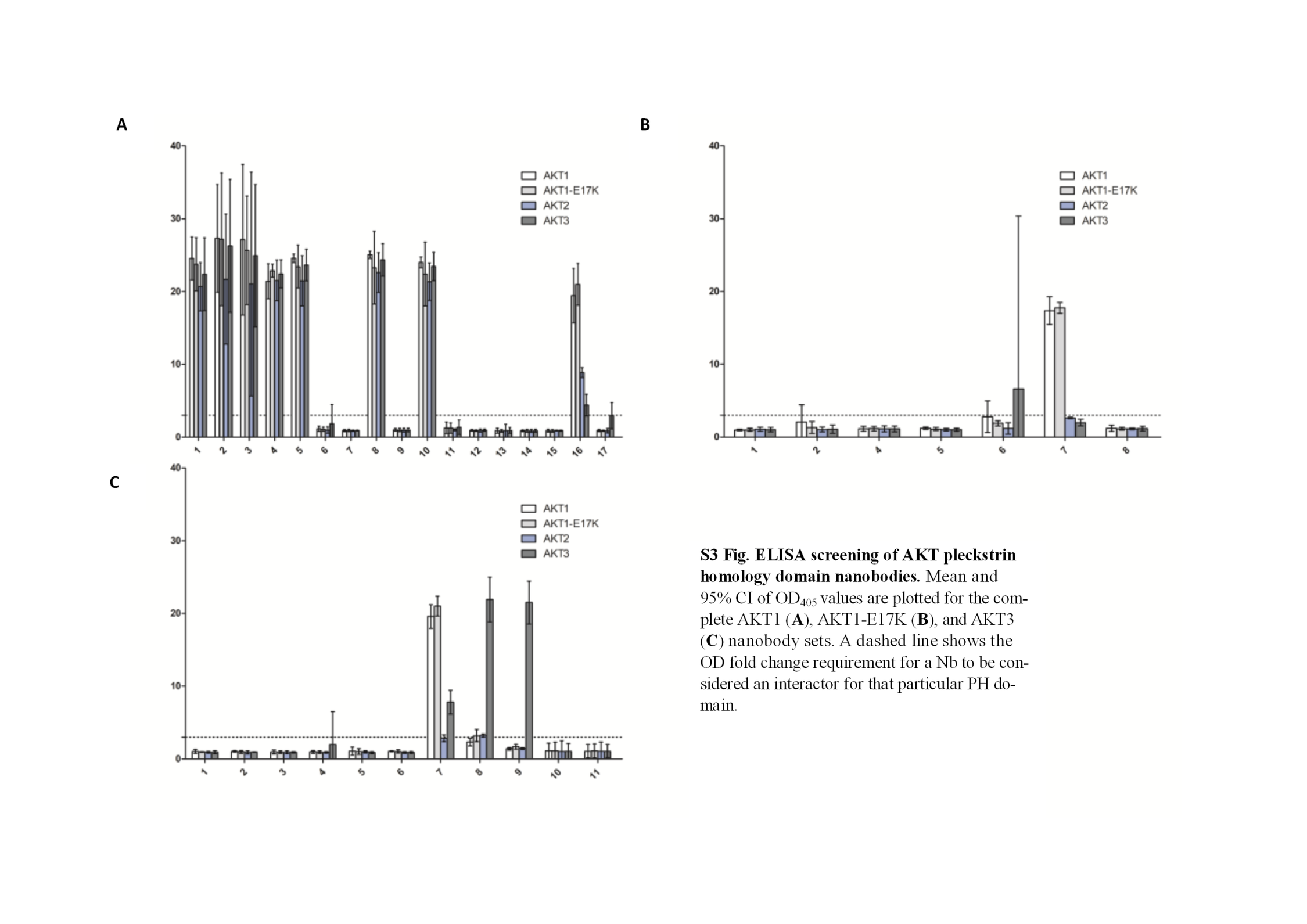

Supplement: S2 Fig — Mean and 95% CI of OD405 values are plotted for the complete AKT1PH (A), AKT1PHE17K (B), and AKT3PH (C) nanobody sets. A dashed line shows the OD fold change requirement for a Nb to be considered an interactor for that particular PH domain. (TIF) [file pone.0240554.s004.tif]

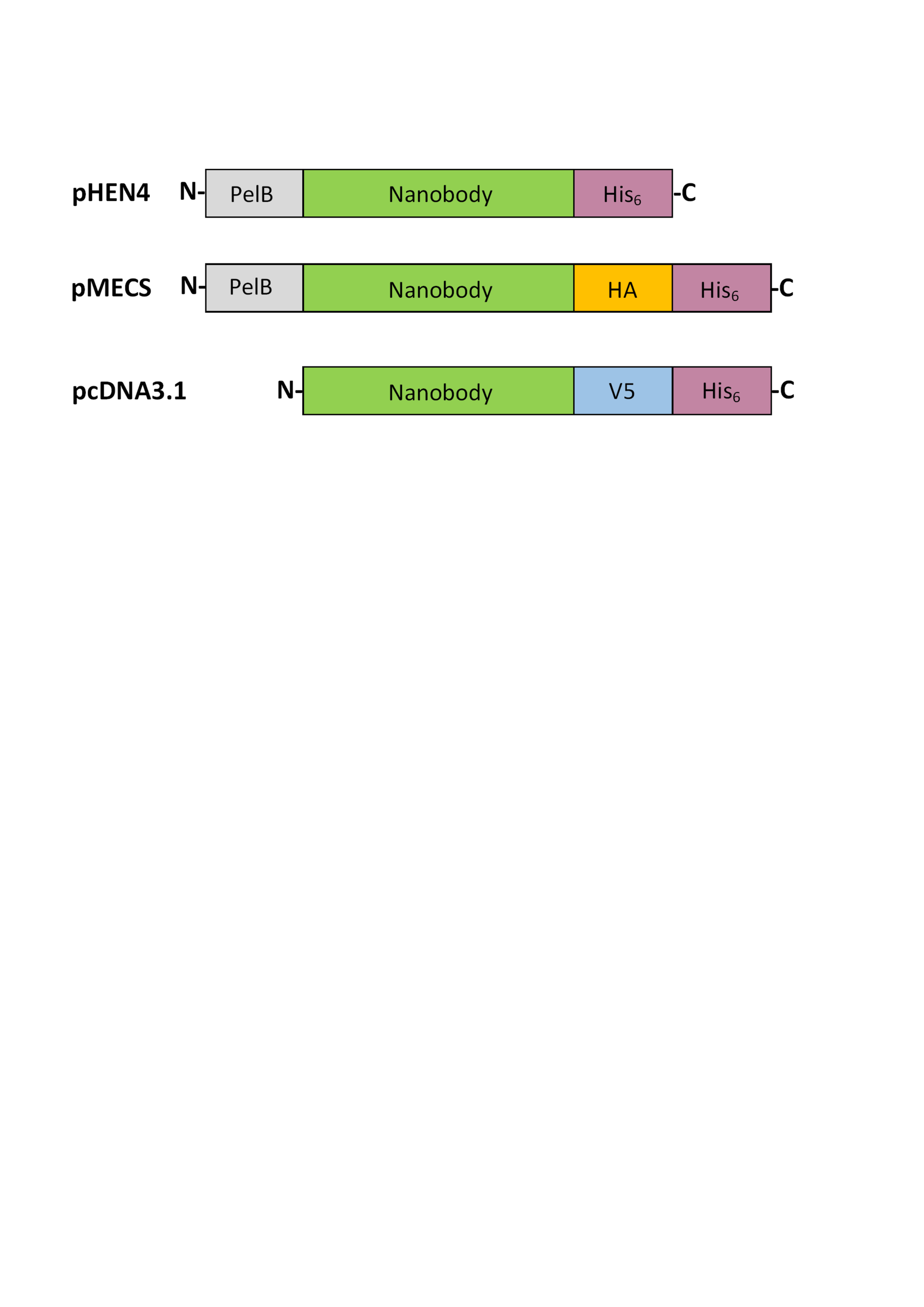

Supplement: S3 Fig — PelB = Signal sequence (22 amino acids) which directs the protein to the E. coli periplasm. This enables the release of recombinantly produced Nbs through osmotic shock. His6 = polyhistidine tag of 6 sequential histidine residues. This tag enables efficient purification of Nbs through IMAC but can also be used for detection. HA = human influenza hemagglutinin tag, a 9 amino acid (YPYDVPDYA) tag. V5 = derived from an epitope found in a virus from the SV5 family, 14 amino acid residues (GKPIPNPLLGLDST). (TIF) [file pone.0240554.s005.tif]

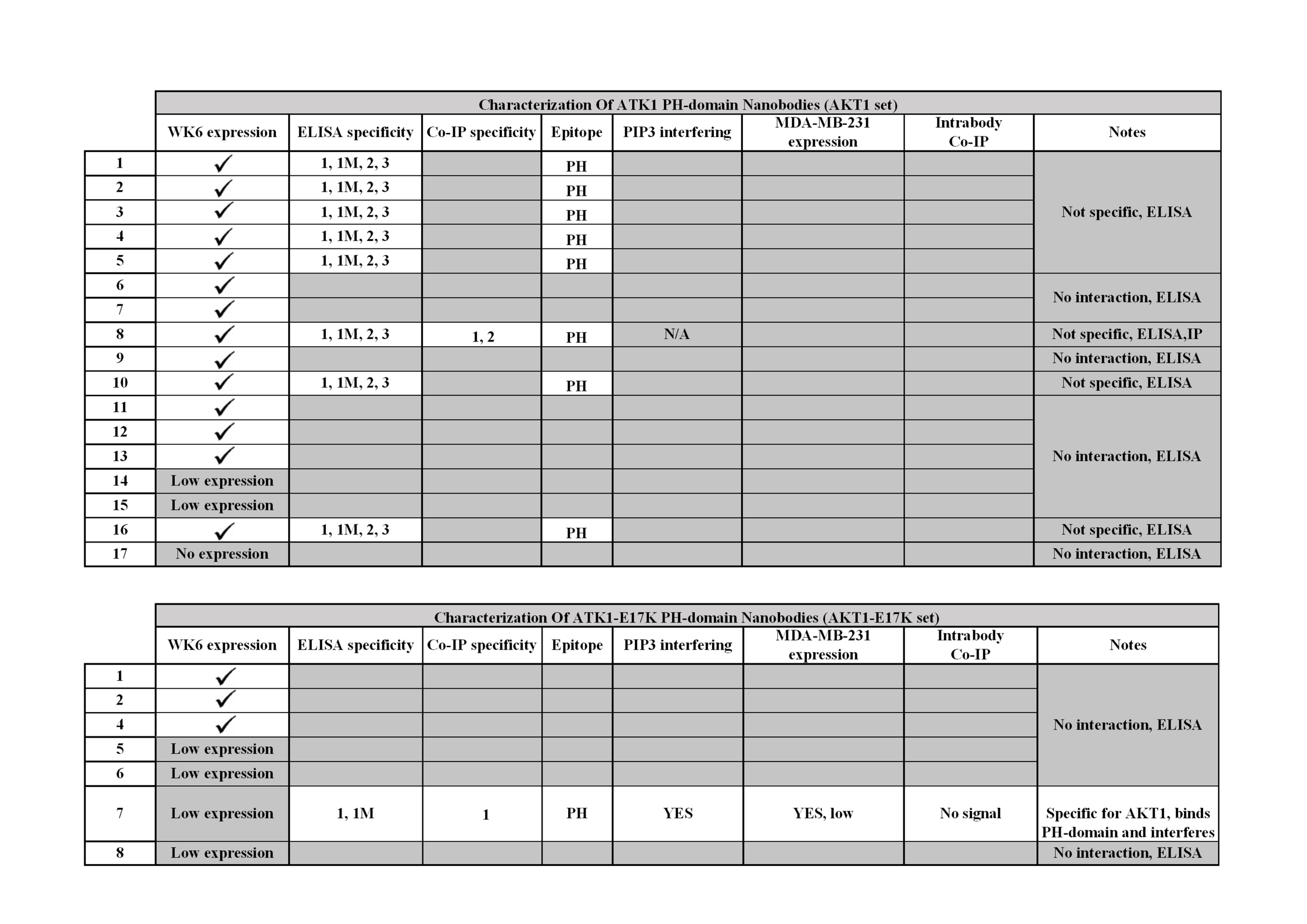

Supplement: S1 Table — Summary of characterization for the AKT1- and AKT1-E17K- nanobody sets. Expression of the Nbs in WK6 E. coli was evaluated though SDS-PAGE & Western blot analysis of crude periplasmatic extracts. AKT isoform-specificity was assessed though both ELISA using recombinant AKT PH-domains (1 = AKT1 PH-domain, 1M = AKT1-E17K PH-domain, 2 = AKT2 PH-domain and 3 = AKT3 PH-domain) and a Co-IP using recombinant Nbs and the endogenous AKT isoforms from MDA-MB-231 crude lysates. A grey filled cell in the ‘ELISA specificity’ column indicates this Nb did not meet the OD fold change requirement for any PH-domain, these Nbs are not included in further experiments. The Co-IP was used as final criteria for specificity. Nbs that interact with an AKT PH-domain can interfere with the PIP3 interaction required for AKT activation. Using PIP3 coated beads, recombinant AKT PH-domains and Nbs we determined AKT1-E17K Nb7 interferes with the interaction of the AKT1 PH-domain AND AKT1-E17K PH-domain with PIP3. Using transient expression a selection of Nbs was expressed in mammalian cells (MDA-MB-231) and evaluated as intrabodies through a Co-IP of endogenous AKT. (TIF) [file pone.0240554.s006.tif]

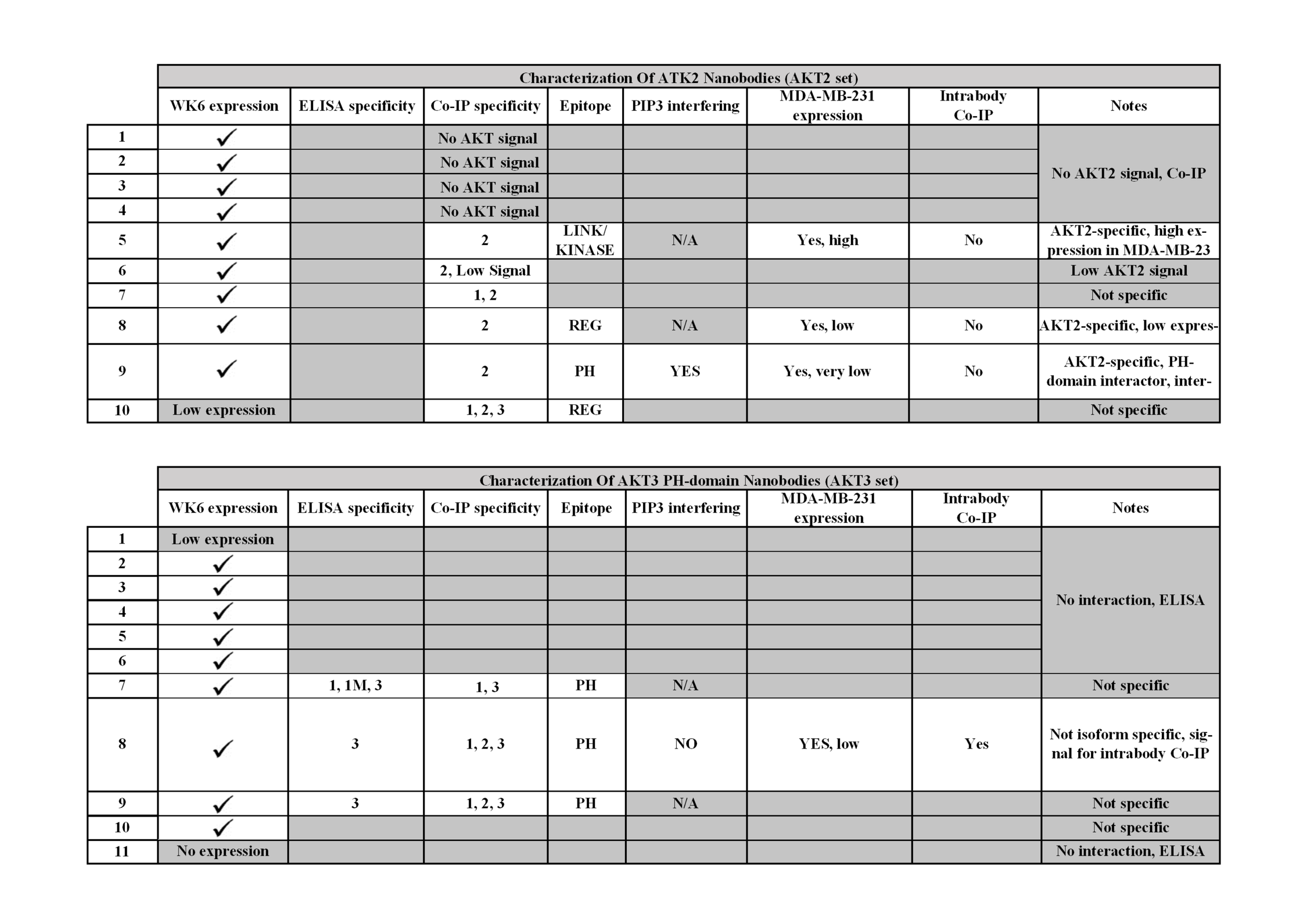

Supplement: S2 Table — Summary of characterization for the AKT2- and AKT3-nanobody sets. Expression of the Nbs in WK6 E. coli was evaluated though SDS-PAGE & Western blot analysis of crude periplasmatic extracts. AKT isoform-specificity was assessed though both ELISA using recombinant AKT PH-domains (1 = AKT1 PH-domain, 1M = AKT1-E17K PH-domain, 2 = AKT2 PH-domain and 3 = AKT3 PH-domain) and a Co-IP using recombinant Nbs and the endogenous AKT isoforms from MDA-MB-231 crude lysates. A grey filled cell in the ‘ELISA specificity’ column indicates this Nb did not meet the OD fold change requirement for any PH-domain, these Nbs are not included in further experiments. The AKT2 Nbs were not included in the ELISA screening. The Co-IP was used as final criteria for specificity. AKT2 Nbs were produced by immunization with full-length AKT2, an ELISA epitope mapping was performed to determine which domain(s) these Nbs bind (PH = pleckstrin homology domain, LINK/KINASE = Linker and kinase domain, REG = regulatory domain). Nbs that interact with an AKT PH-domain can interfere with the PIP3 interaction required for AKT activation. AKT2 Nb9 interferes with the interaction of the AKT2 PH-domain with PIP3. Using transient expression a selection of Nbs was expressed in mammalian cells (MDA-MB-231) and evaluated as intrabodies through a Co-IP of endogenous AKT. (TIF) [file pone.0240554.s007.tif]
